# Abstracts from the 4th Annual Student Medical Summit: Virtual. 6 February 2021

**DOI:** 10.1186/s12919-021-00213-8

**Published:** 2021-05-11

## Abstracts – BMC Proceedings

### A01: An audit of frequent attenders in Cork university hospital emergency department

#### Emer Dight^1^, Conor Deasy^2^

##### ^1^School of Medicine, University College Cork, Co. Cork, Ireland; ^2^Emergency department, Cork University Hospital, Co. Cork, Ireland

###### **Correspondence:** Emer Dight

**Background**

In recent decades overcrowding of hospitals has become a major issue in Ireland. The emergency department, by nature of its walk-in attendees, has been put under increasing pressure. Frequent attenders (FA) have been shown to have increased mortality rates compared to non-frequent attenders (NFA) [1].

The primary aim of this audit was to profile Cork University Hospital’s (CUH) emergency department (ED) FAs and to describe their prevalence. FA were also then compared to NFA where possible. An FA is defined as any patient that attends five or more times per annum.

**Materials and Methods**

A retrospective audit of CUH’s 358 FAs from 1^st^ January to 31^st^ December 2019 was completed. NFA were also analysed for comparative purposes. All data was recorded on Microsoft Excel. The data collected included: arrival date, age, time spent in department, discharge destination and preliminary diagnosis.

**Results**

Approximately 01.1% of patients accounted for 5.7% of attendances in 2019. 358 patients presented a total of 2,565 times to the emergency department. The number of visits per patients ranged from 5 to 68. The average number of visits per patient was seven. The mean age was 56 years. 47% of FA were female and 53% were male. 40% of FA visits were by ambulance compared with 30% by NFAs. FAs were discharged to a ward to receive further care in 43% of cases where NFA went to a ward 29%. FA’s top presenting complaint was ‘unwell adult’ and 4.7% of FA attendances were due to mental illness compared to 0.75% of NFA.

**Conclusion**

This audit was the first of its kind to be done analysing CUH’s FA. Further studies are required to examine measures to reduce FA attendance if appropriate and to reduce the risk of adverse outcomes for this vulnerable group.

**Acknowledgments**

This audit could not have been completed without the aid of my supervisor, Prof Conor Deasy and the Bryan Lynch CUH ED Administration department for assisting in gathering the data.

**Reference**

1. Davison A, Boyle A, Hayhurst C. 44 Quantifying the 5 year mortality of frequent attenders to the emergency department. Emerg Med J [Internet]. 2017 Dec 1 [cited 2020 December 12];34(12):A889–90

### A02: A survey of compliance with the HSE paediatric anaesthesia model of care in Irish hospitals and a local audit of paediatric anaesthesia outcomes

#### Ciara Walsh^1^, John Chandler^2^

##### ^1^School of Medicine, University College Cork, Cork, Ireland; ^2^Department of Anaesthesia, University Hospital Cork, Cork, Ireland

###### **Correspondence:** Ciara Walsh

**Background**

In 2015, the HSE released the Paediatric Anaesthesia Model of Care (PAMoC). It provides a framework for the governance of paediatric anaesthesia in Ireland. The document outlines recommendations pertaining to facilities, training, and structure of the paediatric anaesthesia service. It aims to improve patient outcomes such as postoperative nausea and vomiting, unplanned admissions and fasting times [1]. Thus far, there has been no research investigating the implementation of the PAMoC. This study sought to document the uptake of the PAMoC in non-specialist Irish public hospitals and to assess anaesthesiologists’ attitudes towards this model of care.

**Materials and methods**

All public hospitals in the Republic of Ireland providing a paediatric anaesthesia service, excluding specialist centres operated by Children’s Health Ireland, were invited to participate in this study. An anonymous survey requesting information regarding facilities, training and structure of their paediatric anaesthesia service was sent via email, to assess their compliance with the model of care.

Anonymized data of a random sample of 10% of all children aged 1-5 who had general anaesthesia in 2018 in Cork University Hospital was provided by the Hospital Inpatient Enquiry. Top performance indicators as set out by the PAMoC were collected and compared to international standards. These included fasting times, post-operative nausea/vomiting or unplanned admission after day-case surgery.

**Results**

16 departments responded to the survey (response rate 57%), representing both model 3 and model 4 hospitals. Overall, 93.75% felt the model of care had not meaningfully changed or influenced practice in their department. Only 50% of hospitals have a lead paediatric anaesthesiologist and of these, only 31% lead paediatric anaesthesiologists undertake a paediatric list weekly. In terms of quality improvement, 12 (75%) departments are not routinely recording performance indicators for paediatric anaesthesia.

65 patients were included in the audit. Mean fasting time for this sample was 12 hours. Post-operative nausea and vomiting was identified in 9.7% of the sample. The unplanned admission rate was 18%. In comparison to other specialities, children undergoing orthopaedic surgery were significantly more likely to have an unplanned admission (p<0.003). 73% of unplanned admissions were orthopaedic cases.

**Conclusions**

This study indicates the PAMoC has not been effectively implemented in non-specialist Irish public hospitals, with comparatively high fasting times [2] and unplanned admissions [3] highlighting an area for future study and quality improvement to deliver the best quality anaesthesia care for children in Ireland

**References**

1. HSE Model of Care for Paediatric Anaesthesia. 2015

2. Thomas M, Morrison C, Newton R, Schindler E. Consensus statement on clear fluids fasting for elective pediatric general anesthesia. Pediatric Anesthesia. 2018;28(5):411-414.

3. Royal College of Anaesthetists. Raising the Standard: a compendium of audit recipes. Section 5: Day Surgery Services, Section 9: Paediatrics. 3rd edition, 2012

### A03: A functionalized self-assembling hydrogel for the treatment of osteoarthritis and partial thickness defect of cartilage

#### Alizé Gourrege^1^, Baichuan Wang^1,2^, Hana Alruzaiqi^1^, Zhidao Xia^1^

##### ^1^Centre for Nanohealth, ILS2, Swansea university Medical school, Swansea, SA2 8PP, UK; ^2^Department of Orthopaedics, Union Hospital, Tongji Medical College, Huazhong University of Science and Technology, Wuhan 430022, China

###### **Correspondence:** Alizé Gourrege; Zhidao Xia

**Background**

Cartilage is a tough and flexible connective tissue made up of chondrocytes, which synthesize and turn over the components of the extracellular matrix [1]. It has a role of weight bearing, and act as a cushion and a shock absorber between the bones [2]. Today, 25 million people worldwide suffer from cartilage defect [3]. Once damaged, the cartilage is very unlikely to self-heal due to its avascular nature and the passive diffusion of cells through the matrix [2]. The actual treatments for cartilage damage, including medication, physiotherapy and surgery, do not allow for a complete cure of the tissue and are often seen as both clinically and cost expensive for the patient. There is therefore a need for new treatments which could promote the regeneration of cartilage to a healthy state instead of solely focusing on relieving the symptoms. Tissue engineering appears like a promising option and uses functional scaffolds to recruit endogenous chondrocytes in vivo, at the site of injury [4].

**Materials and methods**

In this study, a new functionalized peptide hydrogel named RA-GF was designed by enriching the bio scaffold PuraMatrix (RADA16) with platelet derived growth factor (PDGF). We hypothesized that RA-GF would better promote the proliferation and cell viability of chondrocytes compared to RADA16 alone. The chondrocytes were isolated and cultured from femoral condyle of bovine knee joints [5,6]. Proliferation tests were performed using RADA16 as control and measurements were taken at day 1,3 and 7. The results were analysed with an ANOVA test to determine any difference between RA-GF and RADA16. Finally, a cytotoxicity test was completed using three different dyes, namely Calcein Acetoxymethyl (Calcein AM), Propidium Iodide (IP) and NucBlue [7]. The number of cells was counted manually based on images obtained from fluorescence microscopy for calculation of percent viability.

**Results**

PDGF significantly increased the proliferation of chondrocytes in vitro (Figure 1). The increase in proliferation and the cell viability seen with RA-GF was not statistically significant compared to RADA16 alone (Figure 2 and 3).

**Conclusions**

RA-GF shows potential as a bio scaffold, however in-depth research over longer periods of time is required to properly evaluate the benefits of this hydrogel in articular cartilage regeneration.

Future work should include the effect of RA-GF on chondrocytes migration, differentiation and expression of chondrogenic related genes in vitro, as well as the in vivo regenerative capacity of RA-GF in induced cartilage defect.

**Acknowledgements**

I would like to express my sincere gratitude to my supervisor Dr Zhidao Xia, who substantially guided me through this research project. Without his valuable advices and help, the writing of this paper would not have been achieved. I would also like to thank the leader of this project, Dr Baichuan Wang. Its invaluable knowledge and experience in regenerative medicine kept me on the right track during this journey. The technical and intellectual contributions of Xiao Li and Hana Alruzaiqi on the ground during laboratory manipulations added up to the quality of this project.

**References**

1. Akkiraju H, Nohe A. Role of Chondrocytes in Cartilage Formation, Progression of Osteoarthritis and Cartilage Regeneration. Journal of Developmental Biology. 2015;3(4):177-192.

2. Sophia Fox A, Bedi A, Rodeo S. The Basic Science of Articular Cartilage: Structure, Composition, and Function. Sports Health: A Multidisciplinary Approach. 2009;1(6):461-468.

3. Damage? W. What is a Cartilage Damage? | Patient Education [Internet]. Patient Education. 2021 [cited 8 January 2021]. Available from: https://cartilage.org/patient/about-cartilage/what-is-cartilage-damage/

4. Tissue Engineering and Regenerative Medicine [Internet]. Nibib.nih.gov. 2021 [cited 8 January 2021]. Available from: https://www.nibib.nih.gov/science-education/science-topics/tissue-engineering-and-regenerative-medicine

5. Vedicherla S, Buckley C. Rapid Chondrocyte Isolation for Tissue Engineering Applications: The Effect of Enzyme Concentration and Temporal Exposure on the Matrix Forming Capacity of Nasal Derived Chondrocytes. BioMed Research International. 2017;2017:1-12.

6. Human Chondrocytes (HC) Culture Protocol [Internet]. Sigma-Aldrich. 2021 [cited 2021 Jan 8]. Available from: https://www.sigmaaldrich.com/technical-documents/protocols/biology/human-chondrocytes.html

7. LIVE/DEAD ® Viability/Cytotoxicity Kit for Mammalian Cells [Internet]. 2005 Dec [cited 2021 Jan 8]. Available from: http://tools.thermofisher.com/content/sfs/manuals/mp03224.pdf

**Fig. 1 (abstract A03).**
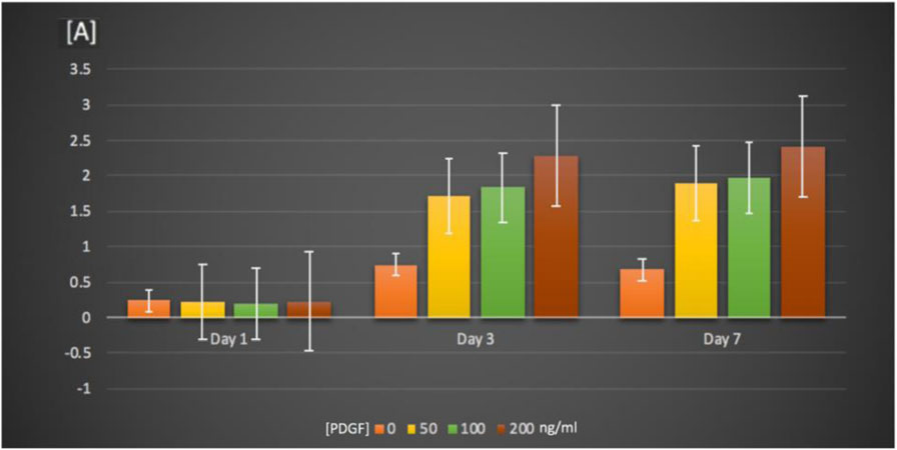
Measurements of the absorbance against the concentration of PDGF alone at day 1, day 3 and day 7

**Fig. 2 (abstract A03).**
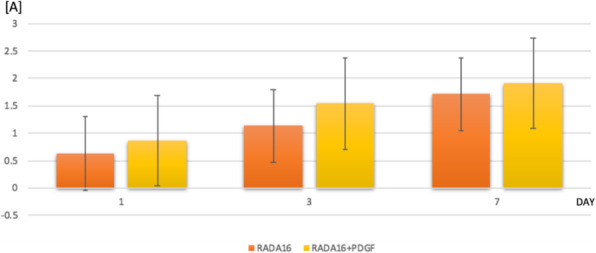
Absorbance of RADA16 and RA-GF in function of the time

**Fig. 3 (abstract A03).**
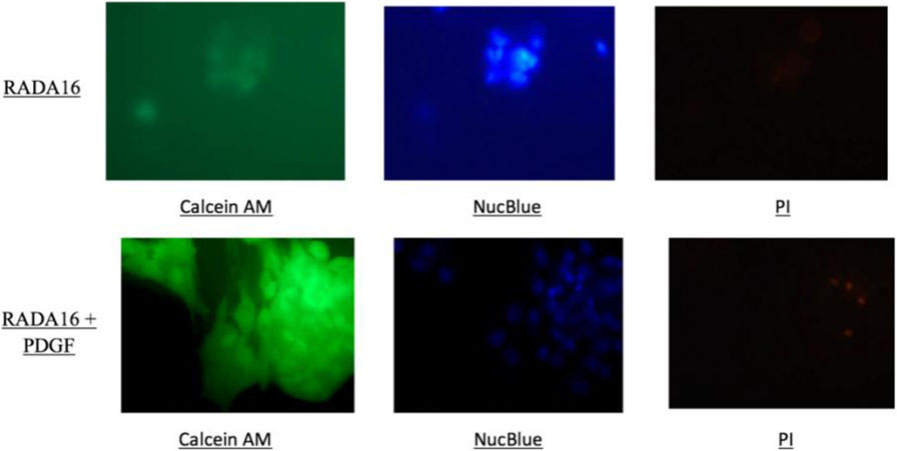
Fluorescent microscope images of articular cartilage cells incubated with RADA16 or RA-GF and stained with Calcein AM, NucBlue or PI

### A04: Educational studies examining knowledge of substance use disorders and career aspirations among medical trainees in an inner-city hospital

#### Luke Gooding^1^, Michee-Ana Hamilton^2^, Huiru Dong^2^, Evan Wood^2,3^, Walter Cullen^1^, Nadia Fairbairn^2,3^, Seonaid Nolan^2^, Jan Klimas^2,4^

##### ^1^School of Medicine, University College Dublin, Health Sciences Centre, Belfield, Dublin 4, Ireland; ^2^British Columbia Centre on Substance Use, University of British Columbia, 400-1045 Howe Street, Vancouver, BC, Canada; ^3^Department of Medicine, University of British Columbia, St. Paul’s Hospital, 608-1081 Burrard Street, Vancouver, BC, Canada; ^4^Department of Family Practice, University of British Columbia, David Strangway Building, 5950 University Blvd, Vancouver, BC, Canada

###### **Correspondence:** Luke Gooding (luke.gooding@ucdconnect.ie)

**Background**

Gaps in addiction medicine training are a reason for poor substance use care in North America [1]. Hospital addiction medicine consult services (AMCS) provide critical medical services, including screening and treatment of substance use disorders (SUD) [2]. While these programs often feature an educational component for medical learners, the impact of AMCS teaching on objective knowledge and career aspirations in addiction medicine has not been well described.

**Materials and Methods**

We report findings from two sequential studies conducted at a large academic hospital in Vancouver, Canada. The first study assessed the impact of an AMCS clinical rotation on medical trainee addiction medicine objective knowledge using an online survey of six true/false questions before and after the rotation. The second study examined the impact of an AMCS rotation on career aspirations using four seven-point Likert-type questions. One-sample t-tests on mean differences (MD) with Benjamini-Hochberg adjustment for multiple comparisons were employed for statistical analyses.

**Results**

Between May 2017 – June 2018, knowledge scores were significantly higher post rotation (MD = 4.78, standard deviation [SD] = 19.5, *p* = 0.034) among 115 medical trainees.

Between July 2018 – July 2019, aspirations to pursue addiction medicine were significantly more favourable post rotation (MD = 3.48, SD = 3.15, *p* < 0.001) among 101 medical trainees.

**Conclusion**

AMCS rotations appear to improve addiction medicine knowledge and aspirations to pursue addiction medicine as a career among medical trainees. Larger-scale evaluations and outcomes research on integrating SUD teaching in these settings will help move the discipline forward.

**Acknowledgements**

The study was supported by the US National Institutes of Health (R25DA037756). This research was undertaken, in part, thanks to funding from the Canada Research Chairs program through a Tier 1 Canada Research Chair in Inner City Medicine that supports Dr. Evan Wood. This project has received funding from the European Union’s Horizon 2020 research and innovation programme under the Marie Skłodowska-Curie grant agreement No 701698. Seonaid Nolan is supported by the Michael Smith Foundation for Health Research and the University of British Columbia’s Steven Diamond Professorship in Addiction Care Innovation. Nadia Fairbairn is supported by an MSFHR/St. Paul’s Foundation Scholar Award.

**References**

1. Ayu AP, Schellekens AF, Iskandar S, Pinxten L, De Jong CA. Effectiveness and Organization of Addiction Medicine Training Across the Globe. Eur Addict Res. 2015;21(5):223-39.

2. Priest KC, McCarty D. Role of the Hospital in the 21st Century Opioid Overdose Epidemic: The Addiction Medicine Consult Service. J Addict Med. 2019;13(2):104-112.

### A05: Systematic literature review identified articles evaluating the positive margin and/or re- operation rate associated with BCS

#### Vikneswaran Raj^1^, Swathica Senthilkumar^1^, Hemali Chauhan^2^, Daniel R Leff^3^

##### ^1^Royal College of Surgeons, School of Medicine, 123 St. Stephen's Green, D02 YN77 Dublin, Ireland; ^2^Clinical Research Fellow, Department of Surgery and Cancer, Imperial College London, St Mary’s Hospital, South Wharf Road, London. W2 1NY; ^3^Reader in Breast Surgery, Departments of BioSurgery and Surgical Technology and Hamlyn Centre for Robotic Surgery at Imperial College London. Honorary Consultant Oncoplastic Breast Surgeon Imperial College Healthcare NHS Trust, St Mary’s Hospital, South Wharf Road, London. W2 1NY

**Background**

Breast cancer is the most common cancer in women with a very high incidence and mortality rate in the UK and Ireland. Breast conserving surgery (BCS) is the most frequently performed procedure for treating women with early stage breast cancer. With a burden of establishing a positive margin in real time, there is an emphasizes on the need of an accurate IMA tool like the iKnife. In order to establish the burden of positive margins a systematic review have been carried out. The systematic review evaluated the effect of DCIS on positive margin. This systematic literature review identified articles evaluating the positive margin and/or re- operation rate associated with BCS [1]. Inclusion and exclusion criteria are as seen in Figure1.

**Materials and methods**

To start, an electronic search was performed on MEDLINE and EMBASE using specific search criteria. Using Covidence, two review authors, independently screened by title and abstract the studies we had identified through the search strategy. Studies were screened and included based on criteria as seen in Figure 1., only clinical studies with data on BCS associated with positive DCIS margins leading to re-excision were incorporated. The remaining papers which were included were then subjected to further screening based on the final full text review. Papers for the full text review were acquired using multiple sources with most papers being obtained from the Endnote software whilst the rest are still being sourced from reaching out to local libraries and journal editors. Alongside this we are finalising a data extraction spreadsheet. Once the full text review is complete, data can then be extracted from each study and incorporated into the spreadsheet. The spreadsheet is being drafted by using different past meta-analysis, similar in nature to our study. We searched for papers from high impact factor publications to ensure the quality and standard of our data extraction. The next step for us is to discuss and finalise the spreadsheet with the final extraction factors which are deemed relevant. To further ensure the quality of the appraisal we are currently searching for different quality scoring systems [2]. Once that is successfully completed, the final step would be to run a meta-analysis to combine the high-quality data extracted.

**Results**

A total of 2,714 studies were imported for screening where 577 duplicates were removed leaving 2,137 studies to be screened. Using Covidence, the studies were filtered, and 1,876 studies were found irrelevant not satisfying the set criteria. The remaining 261 papers are subject to full text screening next before data extraction and meta-analysis can be carried out. Conflicts were discussed and resolved between the review authors. The PRISMA model which is an evidence based minimum set of items for reporting was used as seen in Figure 2. We believe that adhering to the PRIMSA guidelines would reduce potential bias and may improve review quality [3].

**Conclusions**

Currently we are working on the final results of this systematic review. As of now we could only comment on the prevalence of DCIS found at the positive margin and the impact it has on re-excision [4,5]. We hope to gain fruitful insights once the systematic review is completed.

**References**

1. Brouwer de Koning SG, Vrancken Peeters M-JTFD, Jozwiak K, Bhairosing PA, Ruers TJM. Tumor Resection Margin Definitions in Breast-Conserving Surgery: Systematic Review and Meta-analysis of the Current Literature. Clinical breast cancer. 2018;18(4):e595-e600.

2. Bonell C JF, Harden A, et al. Systematic review of the effects of schools and school environment interventions on health: evidence mapping and synthesis. Southampton (UK): NIHR Journals Library; 2013 Jun. (Public Health Research, No. 1.1.) Appendix 6, Data extraction and quality appraisal tables. Available from: https://www.ncbi.nlm.nih.gov/books/NBK262762/.

3. CullisPS, GudlaugsdottirK, AndrewsJ. A systematic review of the quality of conduct and reporting of systematic reviews and meta-analyses in paediatric surgery. PLoS One. 2017;12(4):e0175213.

4. Wei S, Kragel CP, Zhang K, Hameed O. Factors associated with residual disease after initial breast- conserving surgery for ductal carcinoma in situ. Human pathology. 2012;43(7):98693.

5. Houvenaeghel G, Lambaudie E, Bannier M, Rua S, Barrou J, Heinemann M, et al. Positive or close margins: Reoperation rate and second conservative resection or total mastectomy? Cancer Management and Research. 2019;11((Piana) Department of Pathology, Paoli Calmettes Institute, CRCM, CNRS, INSE RM, Marseille 13009, France):2507-16.

**Fig. 1 (abstract A05).**
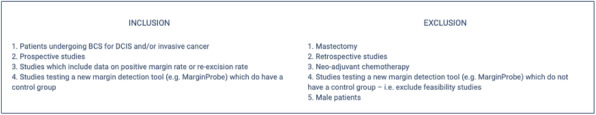
Diagram listing the inclusion and exclusion criteria for the Systematic Review

**Fig. 2 (abstract A05).**
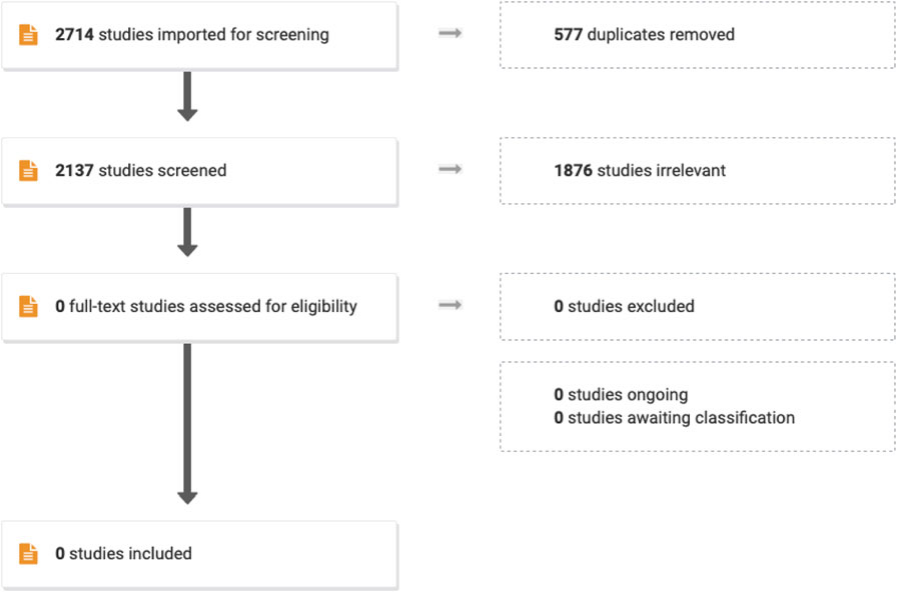
Diagram depicting workflow for the Systematic Review on Covidence

### A06: Diagnostic accuracy of nipple discharge fluid cytology: a meta-analysis and systematic review of the literature

#### Swathica Senthilkumar^2^, Vikneswaran Raj Nagarajan^2^, Natasha Jiwa^1^

##### ^1^Department of Surgery and Cancer, Imperial College London, St Mary’s Hospital, South Wharf Road, London. W2 1NY; ^2^Royal College of Surgeons Ireland, 123 St. Stephen's Green, D02 YN77 Dublin, Ireland

###### **Correspondence:** Swathica Senthilkumar

**Background**

Nipple discharge is the 3rd most frequent complaint of women attending rapid diagnostic breast clinics. Nipple smear cytology remains the single most utilised diagnostic modality for investigation of fluid content, although its diagnostic accuracy remains uncertain. The objective of this paper is to conduct a systematic review and meta-analysis of the diagnostic accuracy of nipple discharge fluid assessment.

**Materials and methods**

This systematic review incorporated Medline, Embase and Scopus databases searches, for studies interrogating the diagnostic data of nipple discharge fluid cytology compared to histopathology gold standard. Data from studies published from 1956- 2019 were analysed. The analysis included 8,648 cytology samples from 59, 991 women. Hierarchical and bivariate models for diagnostic meta-analysis were utilised to attain overall pooled sensitivity and specificity. Sub-group analysis of the diagnostic potential of blood in discharge fluid, as well as imaging modalities was conducted.

**Results**

Of 837 studies retrieved, forty-five studies fulfilled the criteria for review and meta-analysis. Analysis included 8, 648 cytology samples. The diagnostic accuracy meta-analysis of nipple discharge fluid illustrated a sensitivity of 75% [95% CI: 0.74-0.77] and specificity of 87% [95% CI: 0.86-0.87] for benign breast disease, and a sensitivity of 62% [95% CI: 0.53-0.71] and specificity 71% [95% CI: 0.57-0.81] for breast cancer. Furthermore, patients presenting with blood-stained discharge yielded an overall malignancy rate of 58% [0.54-0.60] with a positive predictive value (PPV) of 27% [95% CI: 0.17-0.36]. Pooled ultrasound sensitivity and specificity was 70% [0.60-0.80] and 58% [95% CI: 0.24-0.91]; mammography sensitivity and specificity was 38% [95% CI: 0.23-0.52] and 79% [95% CI: 0.69-0.90] and MRI sensitivity and specificity was 70% [95% CI: 0.61-0.70] and 0.45 [95% CI: 0.20-0.70].

**Conclusions**

Pooled data from all studies encompassing nipple discharge fluid assessment suggests that nipple smear cytology is of limited diagnostic accuracy. Moreover, in patients with only nipple discharge as the presenting symptom, no individual imaging modality has a high enough diagnostic accuracy to exclude carcinoma. Our recommendation is that a tailored approach to diagnosis is required, given variable sensitivities of current available tests.

### A07: Differentiation lineage alters the cytoskeletal and epigenetic response of mesenchymal stem cells to tensile strain

#### Chris Glynn^1^, Stephen D. Thorpe^2^

##### ^1^UCD School of Mechanical and Materials Engineering, University College Dublin, Dublin, Ireland; ^2^UCD School of Medicine, UCD Conway Institute of Biomolecular & Biomedical Research, University College Dublin, Dublin, Ireland

###### **Correspondence:** Chris Glynn

**Background**

Mesenchymal stem cells (MSCs) are widely used for connective tissue regenerative therapies. The application of external mechanical forces to MSCs can initiate and drive fibro-chondrogenic differentiation and is associated with epigenetic modification histone 3 lysine 27 trimethylation (H3K27me3) affiliated with heterochromatin formation [1,2]. The aim of this study is to investigate differentiation-dependent changes in cytoskeletal organisation and nuclear shape in response to 2-D dynamic uniaxial tensile strain.

**Materials and methods**

MSCs were subjected to uniaxial tensile strain of 3% at 1 Hz for 6 hours per day, repeated daily for 3 days in media to encourage osteogenic (OM), adipogenic (AM) or fibro-chondrogenic differentiation alongside control basal media (BM). To explore the role of transforming growth factor-β3 Beta (TGF-β3) in mechanically driven fibro-chondrogenic differentiation, media with (CM+) and without (CM-) TGF-β3 was used. Fluorescently labelled cell images (Figure 1) were processed using a custom MATLAB script to assess filamentous (F-)actin orientation and alignment, nucleus shape and orientation, and H3K27me3 and F-actin intensity pixel intensities within the nucleus.

**Results**

Uniaxial dynamic tensile strain led to alignment of F-actin and nuclei in the direction perpendicular to the direction of stretch in basal, osteogenic and chondrogenic media with TGF-β3 but does not occur in adipogenic or chondrogenic media without TGF-β3.

The correlation between actin fibre and nuclei orientation increased with strain application in basal media but dropped with strain in chondrogenic media with or without TGF-β. This indicates a potential disconnect between the nucleus and cytoskeleton in strained chondrogenic MSCs (Figure 2).

A lack of realignment in chondrogenic media without TGF-β (compared to media with TGF-β) was observed, along with no significant increase in F-actin intensity and a significant reduction in H3K27me3 intensity with stretch. This suggests that TGF-β is necessary for a functional response to stretch.

The combination of strain and differentiation induction led to a reduction in H3K27me3 intensity in all conditions (p < 0.05).

**Conclusion**

Differentiation alters the response of strain. In studying trilineage differentiation of mesenchymal stem cells we see the high dependence of strain response on lineage, suggesting an interplay between biochemical signalling and mechanical signalling.

Future work will investigate the changing nature of cytoskeletal-nucleus connectivity in chondrogenic differentiation as this provides a potential active mechanism whereby the cell regulates strain transfer to the nucleus.

**References**

1. Thorpe, S.D., Lee, D.A. Dynamic regulation of nuclear architecture and mechanics-a rheostatic role for the nucleus in tailoring cellular mechanosensitivity. Nucleus*.* 2017; 8(3):287-300. doi:10.1080/19491034.2017.1285988

2. Heo, S-J., Driscoll, T.P., Thorpe, S.D., et al. Differentiation alters stem cell nuclear architecture, mechanics, and mechanosensitivity. eLife, 2016. doi: 10.7554/eLife.18207

**Fig. 1 (abstract A07).**
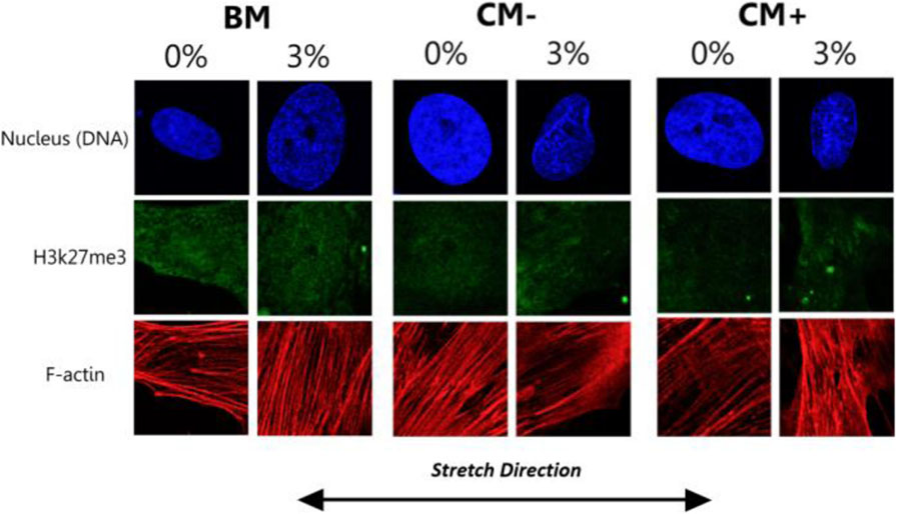
Representative staining across media at different strains

**Fig. 2 (abstract A07).**
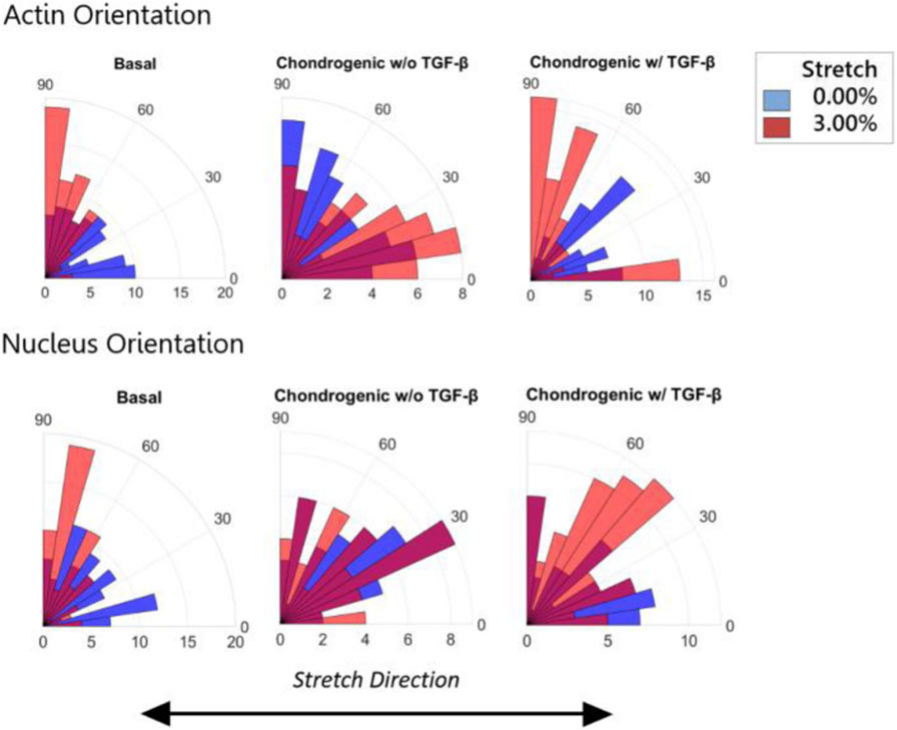
Rose plots for Actin and Nucleus Orientation across Media. Range of angles converted from 180° to 90° to demonstrate contrast to stretch direction

### A08: The impact of wide local excision and sentinel node biopsy on outcomes of patients with melanoma over age 70

#### Kirsten Carpenter^1,2^, Stephanie M Bollard^1,2,3^, Christine S Quinlan^1^, Robert Caulfield^1^, Richard P Hanson^1^, Dylan J Murray^1^, Kevin Cronin^1^, Shirley M Potter^1,2,4^

##### ^1^Department of Plastic & Reconstructive Surgery, Mater Misericordiae University Hospital, Eccles Street, Dublin 7, Dublin, Ireland; ^2^School of Medicine, University College Dublin, Belfield, Dublin 4, Dublin, Ireland; ^3^Charles Institute of Dermatology, University College Dublin, Belfield, Dublin 4, Dublin, Ireland; ^4^Mater Melanoma Group, Mater Misericordiae University Hospital, Eccles Street, Dublin 7, Dublin, Ireland

###### **Correspondence:** Kirsten Carpenter

**Background**

The incidence of melanoma is increasing in the elderly population [1], in whom prevalent comorbidities must be considered perioperatively. Despite significant developments in the adjuvant therapy space, surgery, in the form of wide local excision (WLE) +/- sentinel lymph node biopsy (SLNB), remains the cornerstone of treatment of primary melanoma. This study aims to determine how age and associated comorbid factors influence surgical decision-making and subsequent outcomes for melanoma patients over the age of 70 years.

**Materials and methods**

Data was collected retrospectively for all melanoma patients over the age of 70 treated at a single tertiary referral centre over 10 years. Demographics, comorbidities, diagnosis, surgical management details, disease-free (DFS) and overall survival (OS) were tabulated. The impact of age and comorbidities were analysed.

**Results**

A total of 107 patients met inclusion criteria. The median age was 79.33 (range 70-96) years, and median Breslow Thickness was 1.45mm (range 0.02-22 ). Excisional biopsy only was performed in 15% (n=16), with 85% (n=91) progressing to WLE. Patients who underwent WLE displayed increased DFS (p=0.003), with no impact on OS (p=0.716), and were significantly younger than those who only had excisional biopsy (p=0.003). Of those eligible for SLNB (n=41), 53.7% (n=22) underwent the procedure. Of note, SLNB had no significant impact on DFS (p=0.633) or OS (p=0.222).

**Conclusions**

In the elderly melanoma patient cohort, WLE resulted in improved DFS, but had no effect on OS. If these elderly melanoma patients are suitable surgical candidates, WLE should be offered where possible, in an effort to reduce morbidity from recurrent disease.

**Acknowledgements**

I would like to thank the Mater Misericordiae University Hospital Department of Plastics and Reconstructive Surgery for their guidance throughout all stages of this research.

**Reference**

1. Whiteman DC, Green AC, Olsen CM. The growing burden of invasive melanoma: projections of incidence rates and numbers of new cases in six susceptible populations through 2031. J Invest Dermatol. 2016; 136(6): 1161-1171.

### A09: Infantile spasms in Trisomy 21: A 10 year review of treatment approaches and outcomes in Ireland

#### Kate Flynn^1,2^, Susan Harvey ^1^, Cian Leahy ^1^, Mohamed El Hassan^3^, Jibran Aziz^4^, Caroline Kehoe^5^, Donncha Hanrahan^6^, Sandya Tirupathi^6^, Sally Ann Lynch^2,7^, Mary D King^1,2^, Niamh Lynch^8^, Elizabeth O’Mahony^9^, Niamh McSweeney^4^, Olivia O’Mahony^4^, Nicholas M Allen^5,10^, John C McHugh^2^, David Webb^3^, Mary O’Regan^3^, Amre Shahwan^1^, Declan O’Rourke^1,2^, Brian Lynch^1^, Kathleen M Gorman^1,2^

##### ^1^Department of Neurology and Clinical Neurophysiology, Children’s Health Ireland at Temple Street, Dublin, Ireland; ^2^Department of Neurology, Children’s Health Ireland at Crumlin, Dublin, Ireland; ^3^Department of Paediatrics, Bon Secours Hospital, Cork, Ireland; ^4^Department of Paediatrics, University Hospital Limerick, Limerick, Ireland; ^5^Department of Paediatrics, Cork University Hospital, Cork, Ireland; ^6^Department of Paediatrics, Galway University Hospital, Galway, Ireland; ^7^School of Medicine, National University of Ireland Galway, Galway, Ireland; ^8^Department of Neurology, Royal Belfast Hospital for Sick Children, Belfast, Northern Ireland; ^9^School of Medicine and Medical Science, University College Dublin, Dublin, Ireland; ^10^National Rare Disease Office, Mater Hospital Dublin, Dublin, Ireland

###### **Correspondence:** Kate Flynn

**Background**

Trisomy 21 (T21) or Down syndrome is the most common chromosomal abnormality reported worldwide. The rate of T21 in Ireland is 1 in 411-546 live births, the highest in Europe.^[1]^ Infantile spasms (IS) occur in 0.6-13% of T21 infants and is associated with poorer neurodevelopmental outcomes, increased risk of epilepsy and autism spectrum disorders [2],[3],[4]. The purpose of this review is to identify effective treatment options to assist in management of IS in T21 infants.

**Materials and methods**

A multi-site retrospective 10 year chart review was performed. Inclusion criteria: diagnosis of T21; clinical presentation of IS before 2 years and confirmation of hypsarrhythmia or modified hypsarrhythmia on electroencephalogram (EEG).

**Results**

54 infants were eligible for inclusion in the review. The median age of IS onset (based on parental report) was 201 days (IQR: 156 -242.5 days). The median age of presentation to healthcare setting was 239 days (IQR: 191.5-319.5 days). Initial EEG showed classical hypsarrhythmia in 69% (n=37) with modified hypsarrhythmia in 31% (n=17).

The prescribed first-line medications were: prednisolone (n=20), vigabatrin (n=18), sodium valproate (n=9), combined prednisolone/vigabatrin (n=6) and ACTH (n=1). First-line medication achieved spasm cessation in 44% (n=24). Median of two medications (range: 1-10) were required to achievement of spasm cessation.

The median length of follow-up was 30 months (IQR: 24-49months) Spasm resolution occurred in 85% of infants (n=46). Median time from onset to spasm cessation was 110 days (IQR: 41.8-196days) and from commencing medication to cessation was 18.5 days (IQR: 3.8-117.8days). Two children died. There were ongoing seizures in 24% (13/54) of the cohort, 40% (20/54) were on anti-epileptic medication, and there were developmental concerns in 81%.

Medication side-effects were reported in 17 infants and vigabatrin was associated with 53% (9/17).

**Conclusion**

This review has the largest cohort of T21 patients with IS (n=54) reported to date. Spasm cessation can be achieved in 44% (n=24) after treatment with first medication. Monotherapy prednisolone was the most frequently prescribed first line medication (n=20) achieving spasm cessation in 60% (12/20). Most medications were well tolerated with Vigabatrin accounting for the majority of side effects experienced (46%). Despite high rate of spasm cessation, developmental concerns (83%) and ongoing seizures (24%) were common.

**Acknowledgements**

I would like to thank Dr Kathleen Gorman, Dr Susan Harvey and the neurology department at CHI at Temple Street for their guidance and support in the undertaking of this review, and the preparation of this abstract.

**References**

1. Ni She R, Filan MP., Trisomy 21: incidence and outcomes in the first year, in Ireland today. IMJ. 2014;107(8)

2. Stafstrom C.E., Konkol R.J. Infantile spasms in children with Down syndrome. Developmental Medicine & Child Neurology. 1994;36(7):576-585.

3. Anderson T, Visootsak J, Tapp S. Neurodevelopmental Outcomes in Children with Down syndrome and infantile spasms. Journal of Pediatric Neurology. 2015;13(02):074-077.

4. Sanmaneechai O, Sogawa Y, Silver W, Ballaban-Gil K, Moshé S, Shinnar S. Treatment outcomes of West syndrome in infants with Down syndrome. Pediatric Neurology. 2013;48(1):42-47

### A10: Practices and perspectives with respect to anticoagulation for non-valvular atrial fibrillation in patients on haemodialysis

#### Anna Kelly^1^, Seán Leavey^2^, Michelle M O’Shaughnessy^3^, Ted Fitzgerald^2^

##### ^1^School of Medicine, University College Cork, Cork, Ireland; ^2^Department of Nephrology, University Hospital Waterford, Waterford, Ireland; ^3^ Department of Renal Medicine, Cork University Hospital, Cork, Ireland

###### **Correspondence:** Anna Kelly

**Background**

Non-Valvular Atrial Fibrillation (NVAF) is the most common cardiac arrhythmia and can result in ischaemic stroke [1]. Compared to the general population, patients with kidney failure receiving haemodialysis have a higher incidence of NVAF and stroke, but also of major bleeding [2]. Studies examining the risk-to-benefit ratio of oral anticoagulation (OAC) for NVAF in patients receiving haemodialysis have produced inconclusive findings [3]. We investigated patient and physician perspectives with respect to the risks and benefits of OAC for NVAF in patients receiving maintenance haemodialysis.

**Materials and Methods**

We screened all patients scheduled to attend any of two hospital-based and one community-based dialysis clinic in the month of October 2019 for a diagnosis of NVAF. We collected demographic, comorbidity, dialysis prescription, and medication data for all identified cases. Patients were interviewed regarding their understanding of NVAF and associated stroke and bleeding risks. Separately, physician members of the Irish Nephrology Society were surveyed regarding their perspectives on NVAF management in haemodialysis patients and perceived risk-benefit of OAC in six hypothetical cases. T-test and Chi-square tests were used for univariate analyses. Two-way repeated measures ANOVA was used to examine variation within and across case vignettes with respect to perceived risk-benefit of OAC.

**Results**

We identified 41 patients (17% of those screened) with NVAF. 18 (44%) of these patients were prescribed OAC (11 warfarin, 7 apixiban). OAC use was positively associated with heart failure (p=0.01), higher BMI (p=0.01) and CHA_2_DS_2_-VASc scores ≥ 3 (p<0.05). Otherwise, there were no meanginful clinical differences, and no statistically significant differences, in the characteristics of those receiving vs. not receiving OAC. 27% of surveyed patients reported awareness of stroke risk in NVAF and 61% of those prescribed OAC were aware of the bleeding risk.

21 physicians responded to the survey, a response rate of 17.8%. Uncertainty regarding NVAF management existed, with 90% asserting clinical equipoise surrounding OAC therapy. Varying the stroke, bleeding, and falls risk in the 6 hypothetical cases did significantly influence risk-benefit perceptions and likelihood to prescribe OAC (p<0.01). However, irrespective of baseline risk-benefit perception, physicians were positively biased towards initiating OAC, and further biased towards continuing OAC (if already prescribed) (p<0.01).

**Conclusions**

A paucity of evidence regarding the relative risks and benefits of OAC for NVAF in patients on dialysis has led to inconsistent and uncertain physician practice patterns. There is urgent need for randomized controlled trials of OAC for NVAF in patients on dialysis.

**References**

1. Go AS, Hylek EM, Phillips KA, Chang Y, Henault LE, Selby JV, Singer DE. Prevalence of diagnosed atrial fibrillation in adults: national implications for rhythm management and stroke prevention: the AnTicoagulation and Risk Factors in Atrial Fibrillation (ATRIA) Study. JAMA. 2001; 285(18):2370-5.

2. De Vriese AS, Caluwé R, Raggi P. The atrial fibrillation conundrum in dialysis patients. Am Heart J. 2016; 174:111-9.

3. Van Zyl M, Abdullah HM, Noseworthy PA, Siontis KC. Stroke Prophylaxis in patients with atrial fibrillation and end-stage renal disease. J Clin Med. 2020; 123:1-15

### A11: Where are all the children? Exploring the impact of COVID-19 related lockdown restrictions on mental health presentations to an Irish paediatric emergency department

#### Brigid Kemerer^1^, Sarah Casey^2^, Ian McClelland^2^, Elizabeth Barrett^1,2^

##### ^1^UCD School of Medicine, University College Dublin, Belfield, Dublin 4, Ireland; ^2^Department of Liaison Psychiatry, Children’s Health Ireland at Temple Street, Dublin 1, Ireland

###### **Correspondence:** Elizabeth Barrett

**Background**

On March 12^th^, 2020, the Irish government implemented disease containment measures due to the COVID-19 pandemic leading to widespread social isolation, school closures and changes in daily routine [1]. While older individuals are considered most at risk physically, evidence from the H1N1 and SARS epidemics showed that approximately 33.0% of quarantined children availed of mental health services due to anxiety and adjustment disorders highlighting the potential impact of a pandemic on paediatric mental health [2]. A prospective study was conducted exploring the impact of COVID-19 and the first national lockdown on paediatric mental health presentations to the Emergency Department (ED) at Temple Street Children’s University Hospital (TSCUH).

**Materials and methods**

ED mental health presentations from March-April 2019 (n=79) and 2020 (n=60) were reviewed. Multiple variables, including reason for presentation, diagnosis and presence of self-harm (SH) and suicidal intent, were prospectively gathered for 2020 cases and compared to 2019 presentations. Descriptive analyses of clinical findings using T tests, chi-square tests and one-way ANOVA tests were performed as appropriate and statistical significance was considered with p < 0.05. Ethical exemption was granted by the chair of the local ethics committee.

**Results**

ED mental health presentations reduced by 24.1% in 2020 with a proportional increase in SH presentations (+8.6% (x^2^=1.03, p=0.31)) and reported suicidal intent (+39.2% (x^2^=15.04, p<0.001)). Proportions of re-presentations to ED increased in 2020, noting a 17.5% (x^2^=6.70, p=0.01) increase in those previously presenting with SH and a 43.6% (x^2^=25.88, p<0.001) increase in those previously presenting for other indications. Results show a 7.6% rise in attendance from care settings, with all children in care settings who presented to TSCUH ED in April 2020 citing COVID-19 to trigger ED presentation. More children presented with a family history of SH (+14.9% (x^2^=4.75, p= 0.03)), those already prescribed psychotropic medication (+30.7% (x^2^=23.31, p<0.001)) and/or attending psychotherapy (33.3% (x^2^=15.01, p<0.001)) in 2020. A 22.1% increase in ED presentations with two or more diagnoses was observed in 2020 as well as changes in factors precipitating ED attendance such as arguments with family members (+9.8%), social isolation (+7.2%) and school pressure (-18.0%).

**Conclusions**

Our findings show fewer ED presentations during lockdown. Those who present are often high risk and known to services. Further research is needed to explore reasons for this in order to proactively manage vulnerable individuals in the community through effective service planning.

**References**

1. The Irish Times. Coronavirus: How it has affected Ireland day-by-day. March 19 2020. Available at:https://www.irishtimes.com/news/health/coronavirus-how-it-has-affected-ireland-day-by-day-1.4206691 [3 August 2020]

2. Sprang G. and Silman M. Posttraumatic stress disorder in parents and youth after health-related disasters. Disaster Medicine and Public Health Preparedness. 2013; 7(1): 105-110.

### A12: Identifying SARS-CoV2 transmission cluster category: An analysis of country government database

#### Basem Fouda^1,2,⊕^, Ha Pham Bich Tram^2,3,⊕^ , Omar Mohamed Makram^2,4,5,⊕^, Abdelrahman Sherif Abdalla^2,6,⊕^, Tushar Singh^1,2,⊕^, I-Chun Hung^2,⊕^, Lina Hemmeda^2,7^, Majd Alahmar^2,8^, Akshay Raut^2,9^, Ahmed Sallam ElHawary^2,10^, Dina Awad^2,11^, Nguyen Tien Huy^12,13^

##### ^1^School of Medicine, Trinity College Dublin, Ireland; ^2^Online Research Club, Nagasaki, Japan; ^3^The VNUK Institute for Research and Executive Education, The University of Da Nang, Da Nang, Vietnam; ^4^Faculty of Medicine, October 6 University, Giza, Egypt; ^5^Faculty of Public Health and Policy, London School of Hygiene and Tropical Medicine, London, United Kingdom; ^6^Faculty of Medicine, El-Minia University, El-Minya, Egypt; ^7^Faculty of Medicine, University of Khartoum, Khartoum, Sudan; ^8^Faculty of Medicine, Mansoura University, Mansoura, Egypt; ^9^Rajarshee Chhatrapati Shahu Maharaj Government Medical College, Kolhapur 41002, Maharashtra, India; ^10^Faculty of Medicine, South Valley University, Qena City, Egypt; ^11^Alexandria Faculty of Medicine, Alexandria University, Alexandria, Egypt; ^12^Institute of Research and Development, Duy Tan University, Da Nang 550000, Vietnam; ^13^School of Tropical Medicine and Global Health, Nagasaki University, 1-12-4 Sakamoto, Nagasaki 852-8523, Japan

###### **Correspondence:** Basem Fouda; Tushar Singh

^⊕^Authors equally contributed to the work; i.e., the first 6 authors are co-first authors who contributed to this research equally with regards to time and effort dedicated.

**Background**

This study seeks to identify settings that are more prone to the transmission of COVID-19, which can provide insight regarding the opening and closure of such settings as well as monitoring and testing. As a result, this study can assist governments in prioritizing control measures when tackling possible future waves of the pandemic and future pandemics of a similar nature.

**Materials and methods**

Following a comprehensive review of the relevant literature and media articles, extraction of the cluster data of eight countries was performed by way of hand searching of reputable databases. The following data was extracted and arranged in an accessible online sheet: The total number of clusters and cases for each cluster type, the total number of cases in the country, date and source of the data collection. The cluster types were divided into 10 main types, with subcategories for specified types. Each country had 2 members assigned for data validation and review.

**Results**

Among the eight included countries, we have found 3905 clusters and a total number of 1907974 patients. Indoor settings (mass accommodation and residential facilities) comprised the highest number of both number of clusters (3313/3905) and infected patients (1836870/1907974), while the outdoor ones comprised 592 clusters and 71104 patients. Mass accommodation was associated with the highest number of cases in 5 of the 8 countries. Social events and residential settings were responsible for the highest number of cases in South Korea and Malaysia, respectively. In the USA, workplace facilities have reported 165 clusters of infection including 122 food production facilities.

**Conclusions**

As lockdowns pose a dilemma to governments worldwide due to the widespread effects of these measures, obtaining appropriate information concerning the transmissibility and the behavior of the disease is crucial in order to guide the removal of lockdowns across different fields and regions.

### A13: Cross-reactivity and conservation of T-cell epitopes across the human coronaviruses

#### Joseph J. Cronin^1^, Damien Farrell^2^

##### ^1^UCD School of Medicine, Dublin, Ireland; ^2^UCD School of Veterinary Medicine, Dublin, Ireland

**Background**

As the COVID-19 pandemic continues to dominate the globe and cause substantial mortality and morbidity, new research has put the spotlight on the T-cell response to SARS-cov-2. With particular scrutiny and import being placed on the suggested cross-reactivity of memory T-cells specific to a common-cold coronavirus having heterologous immunity to SARS-cov-2.

**Materials and Methods**

Using an epitope prediction software, NetMHCpan, a list of the likely MHCII epitopes for CD4 T-cells, representing the 8 most common alleles, was generated for the SARS-cov-2 spike protein. Following the methods of Mateus et al, NetMHCpan was configured to predict 15-mer epitopes, where only a core 9 amino acids had to be conserved (Table 1) [1]. Additionally, a sequence alignment of the spike protein genome for these viruses was performed using Jalview to manually assess for conservation and a literature review of relevant papers on T-cell cross-reactivity were performed (Figure 1).

**Results**

Out of 92 SARS-cov-2 epitope predictions, 2 epitopes were conserved across the board with SARS and the four common-cold coronaviruses, and 8 epitopes had varying degrees of conservation with different computations of the 6 coronaviruses.

**Conclusions**

These conserved epitopes across the human coronavirus family are significant in that they contribute to the growing body of evidence of human coronavirus memory T-cells displaying heterologous immunity to SARS-cov-2. The results underscore the need for further research, but also these results and how they fit in with the previous literature, informs understanding of the immune response to COVID-19 and future therapeutic designs, and offers an explanation for the variability of symptoms of COVID-19 symptoms [1, 2].

**References**

1. Mateus, Jose, et al. ‘Selective and Cross-Reactive SARS-CoV-2 T Cell Epitopes in Unexposed Humans’. *Science*, Aug. 2020. *science.sciencemag.org*, doi:10.1126/science.abd3871.

2. Sekine, Takuya, et al. ‘Robust T Cell Immunity in Convalescent Individuals with Asymptomatic or Mild COVID-19’. *BioRxiv*, June 2020, p. 2020.06.29.174888. *www.biorxiv.org*, doi:10.1101/2020.06.29.174888.

**Table 1 (abstract A13).** Top 12 predicted epitopes and corresponding HCoV homolog % Identity

peptide	pos	name	alleles	core	median_rank	sars	229E	NL63	OC43	HKU1	total
**AEVQIDRLITGRLQS**	**988**	**GU280_gp02**	**8**	**IDRLITGRL**	**246.5**	**0.85**	**0.69**	**0.69**	**0.77**	**0.77**	**3.77**
**VEAEVQIDRLITGRL**	**986**	**GU280_gp02**	**5**	**VQIDRLITG**	**456**	**0.85**	**0.69**	**0.69**	**0.77**	**0.77**	**3.77**
**KLQDVVNQNAQALNT**	**946**	**GU280_gp02**	**4**	**VVNQNAQAL**	**607**	**0.85**	**0.62**	**0.69**	**0**	**0.69**	**2.85**
**GAISSVLNDILSRLD**	**970**	**GU280_gp02**	**3**	**VLNDILSRL**	**1019**	**0.85**	**0**	**0**	**0.69**	**0.69**	**2.23**
**FIEDLLFNKVTLADA**	**816**	**GU280_gp02**	**6**	**LLFNKVTLA**	**457**	**0.85**	**0**	**0**	**0.69**	**0.69**	**2.23**
**KRSFIEDLLFNKVTL**	**813**	**GU280_gp02**	**4**	**FIEDLLFNK**	**834**	**0.85**	**0**	**0**	**0.69**	**0.69**	**2.23**
**DRLITGRLQSLQTYV**	**993**	**GU280_gp02**	**7**	**LITGRLQSL**	**794**	**0.85**	**0.69**	**0.69**	**0**	**0**	**2.23**
**ALNTLVKQLSSNFGA**	**957**	**GU280_gp02**	**8**	**VKQLSSNFG**	**255**	**0.85**	**0**	**0**	**0.69**	**0**	**1.54**
**TQNVLYENQKLIANQ**	**911**	**GU280_gp02**	**3**	**VLYENQKLI**	**466**	**0.85**	**0**	**0**	**0.69**	**0**	**1.54**
**NQKLIANQFNSAIGK**	**918**	**GU280_gp02**	**6**	**LIANQFNSA**	**560**	**0.77**	**0**	**0**	**0.69**	**0**	**1.46**
**FGAISSVLNDILSRL**	**969**	**GU280_gp02**	**5**	**FGAISSVLN**	**278**	**0.85**	**0**	**0**	**0**	**0**	**0.85**
**DDSEPVLKGVKLHYT**	**1258**	**GU280_gp02**	**5**	**VLKGVKLHY**	**723**	**0.85**	**0**	**0**	**0**	**0**	**0.85**

**Fig. 1 (abstract A13).**
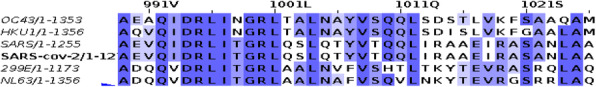
Sequence alignment of S proteins of the HCoVS performed in Jalview

### A14: The pathogenicity of *Helicobacter Pylori* in Parkinson’s Disease

#### Kevin Crishan^1^, Justin McCarthy^2^

##### ^1^School of Medicine, University College Cork, Cork, Ireland; ^2^School of Biochemistry & Cell Biology, University College Cork, Cork, Ireland

###### **Correspondence:** Kevin Crishan

**Background**

Parkinson’s disease (PD) is the second most common neurodegenerative disease after Alzheimer’s disease, but the exact etiology of PD remains unclear [1]. Recent studies have shown that GI symptoms serve as a prodrome to PD and this suggests that *Helicobacter pylori* may play a role in infection among PD patients, but detection and eradication of *H. pylori* are not part of current PD management.

**Methods**

Articles were selected from MEDLINE and EMBASE databases according to inclusion and exclusion criteria for this meta-analysis. The prevalence of *H. pylori* infection in PD, its relationship with unified Parkinson’s disease rating scale (UPDRS) scores and gut SNCA gene expression in cases and controls were analyzed using odds ratios (OR) and standardized mean differences (SMD) with 95% confidence intervals (CI). Fixed and random-effects models were applied. All statistical analyses were performed with the Review Manager (RevMan) Version 5.4 software suite.

**Results**

Eleven studies were included in the first meta-analysis (5039 PD cases, 23194 controls) where H. pylori infection was more prevalent in PD patients [OR (95% CI): 1.46 (1.26, 1.68), Pz < 0.00001]. Five studies which included UPDRS scores showed a significant association between *H. pylori* infection and mean UPDRS scores [SMD (95% CI): 0.27 (0.02, 0.52), Pz = 0.03]. SNCA gene expression was also significantly higher among *H. pylori* patients [SMD (95% CI): 0.89(0.09, 1.69), Pz = 0.03].

**Conclusion**

A higher prevalence of *H. pylori* was found among PD patients with a consistently lower UPDRS score among the healthy controls shows not only the increased risk among the infected cohort but how it worsens motor function. Furthermore, the significance of SNCA expression in gut biopsies of *H. pylori* infected patients suggests its importance in the management of PD and potentially as a screening tool.

**Acknowledgements**

This work was funded by the Research and Postgraduate Affairs Committee, UCC

**Reference**

1. Tysnes O, Storstein A. Epidemiology of Parkinson’s disease. Journal of Neural Transmission. 2017; 124(8):901-905.

### A15: Harnessing artificial intelligence in cardiac rehabilitation, a systematic review

#### Sara Sotirakos^1^, Basem Fouda^1^, Noor Adeebah M. Razif^1^, Cormac Mulhall^1^, Aisling O’Byrne^1^, Bridget Moran^1^, Ruairi Connolly^1,2^

##### ^1^Trinity College Dublin, School of Medicine, Dublin 2, Ireland; ^2^National Rehabilitation Hospital, Dublin, Ireland

###### **Correspondence:** Basem Fouda (bfouda@tcd.ie)

**Background**

Clinical tools based on artificial intelligence have shown a lot of potential for heart disease treatment, diagnosis and monitoring. Artificial intelligence (AI) is defined as “the concept that machines can be improved to assume some capabilities normally thought to be like human intelligence such as learning, adapting, self-correction, etc.” [1]. In this review, we explore how AI is currently being utilised for the purpose of cardiac rehabilitation.

**Materials and methods**

The libraries PubMed, Medline, Embase, Cochrane, and Scopus were searched for suitable papers using search terms for heart disease, artificial intelligence and rehabilitation. Table 1 contains a summary of major inclusion and exclusion criteria.

**Results**

The search returned 156 studies. After screening according to our inclusion criteria, 8 studies were included in our review.

*Smart Watches*

Mobile devices like the Apple Watch or Fitbit Charge HR 2 have demonstrated their potential for health monitoring and making earlier diagnosis. This enables the prevention of major adverse cardiac events and therefore, a reduction in hospitalization rates.

*Home-Based Rehabilitation and Monitoring*

Home-based interventions consist of home-based exercise protocols in combination with a digital health system used for delivering the protocol and for patients to report their progress. The use of such tools can help patients lose weight and improves body composition, acting as a form of secondary prevention [2]. In addition, home-based cardiac rehabilitation programs can greatly improve accessibility for patients.

*Decision Making Support*

AI-based technologies also have the potential to be an aid to the physician. For patients with ambiguous or unclear symptoms, the use of a neuro-fuzzy model can offer doctors decision-making support when it comes to prescribing different therapies [3]. Such models can help reduce physician error and therefore, improve patient outcomes.

**Conclusions**

AI based technologies are a very promising advancement in the field of cardiac rehabilitation. While new, they have demonstrated the potential to increase patients’ autonomy through home-based interventions, improve patient outcomes by alerting them to seek timely medical care, and guide physicians through the decision-making process.

**References**

1. Kok JN, Boers E, Kosters WA, Van der Putten P, Poel M. Artificial intelligence: definition, trends, techniques, and cases. Artificial intelligence. 2009;1:1-20.

2. Widmer RJ, Allison TG, Lennon R, Lopez-Jimenez F, Lerman LO, Lerman A. Digital health intervention during cardiac rehabilitation: A randomized controlled trial. Am Heart J. 2017;188:65-72.

3. Obot OU, Uzoka FM, Akinyokun OC, Andy JJ. A neuro-fuzzy decision support model for therapy of heart failure. 2014;6(4):319-44.

**Table 1 (abstract A15).** Major inclusion and exclusion criteria

**Inclusion**	Participants	- Heart disease - Eligible for cardiac rehabilitation
	Intervention	- AI-based technology
	Control	- Usual care cardiac rehabilitation
	Outcome(s)	- Physical activity (fitness measures, adherence) - Diet (adherence) - Blood pressure - Recurrence / Hospitalisation - Quality of Life - Acceptance / Satisfaction
**Exclusion**	Others	- Non-English language papers

### A16: Can the collapse of the scalp hair follicle pigmentary unit with age (canities) provide insights into how melanocyte death could be induced in melanoma?

#### Halin Buruno^1^, Daniel Johnston^1,2^, Desmond J. Tobin^1,2^

##### ^1^UCD School of Medicine, University College Dublin, Belfield, Dublin 4, Ireland; ^2^UCD Charles Institute of Dermatology, UCD School of Medicine, Belfield, Dublin 4, Ireland

###### **Correspondence:** Halin Buruno

**Background**

Melanoma, a potentially deadly skin cancer is increasing incidence worldwide. It develops from melanocytes located preferentially in the pigmented epidermis rather than the pigmented hair follicle(HF) epithelium [1]. In greying HFs the aging follicular melanin unit has been associated with increased melanocyte death by apoptosis perhaps triggered by uncontrolled oxidative stress(OS). Ataxia Telangiectasia Mutated(ATM) is a protein kinase that can sense DNA damage and OS, however its activation mechanisms in skin are little understood [2]. We aimed to investigate the relationship of melanocyte death (in greying HFs) and ATM sensing of OS.

**Materials and methods**

Human haired scalp tissue (n=7,all male[25-73yrs,mean=46,4>40yrs]) was ethically obtained from The Charles Institute via Hair Restoration Blackrock. Tissue sections (5-10μm) were cut and a double immunohistochemistry assay was performed using a melanocyte lineage marker (Nkibeteb) and antibodies to ATM and phospho-ATM. Images were prepared using CellSense and ImageJ.

**Results**

Melanocyte number decreased in greying HFs as pigmentation decreased (Figure 1). Nuclear ATM was expressed in the HF bulbar melanocytes and in some fibroblast cells of the dermal papilla of the HF bulb, but not in melanocytes of the more superficial and UV exposed epidermis (Figure 2,3). By contrast, phospho-ATM was expressed cytoplasmically in the keratinocytes of the epidermis and of the upper HF (Figure 3).

**Conclusions**

Results confirmed melanocyte depletion in human canities-affected HF and also suggested a protective role of ATM to OS in HF bulbs that retained pigmented melanocytes even at significant age. A potential melanoma intervention strategy may be to modulate ATM kinase expression in melanoma cells to make these cells more susceptible to a canities-like deletion [1,2].

**Acknowledgements**

I would like to acknowledge the opportunity, time and guidance given from Professor Desmond Tobin and members of the Charles Institute of Dermatology.

**References**

1. Bedogni B, Paus R. Hair(y) Matters in Melanoma Biology. Trends in Molecular Medicine. 2020;26(5):441-449.

2. Kozlov S, Graham M, Jakob B, Tobias F, Kijas A, Tanuji M et al. Autophosphorylation and ATM Activation. Journal of Biological Chemistry. 2010;286(11):9107-9119.

**Fig. 1 (abstract A16).**
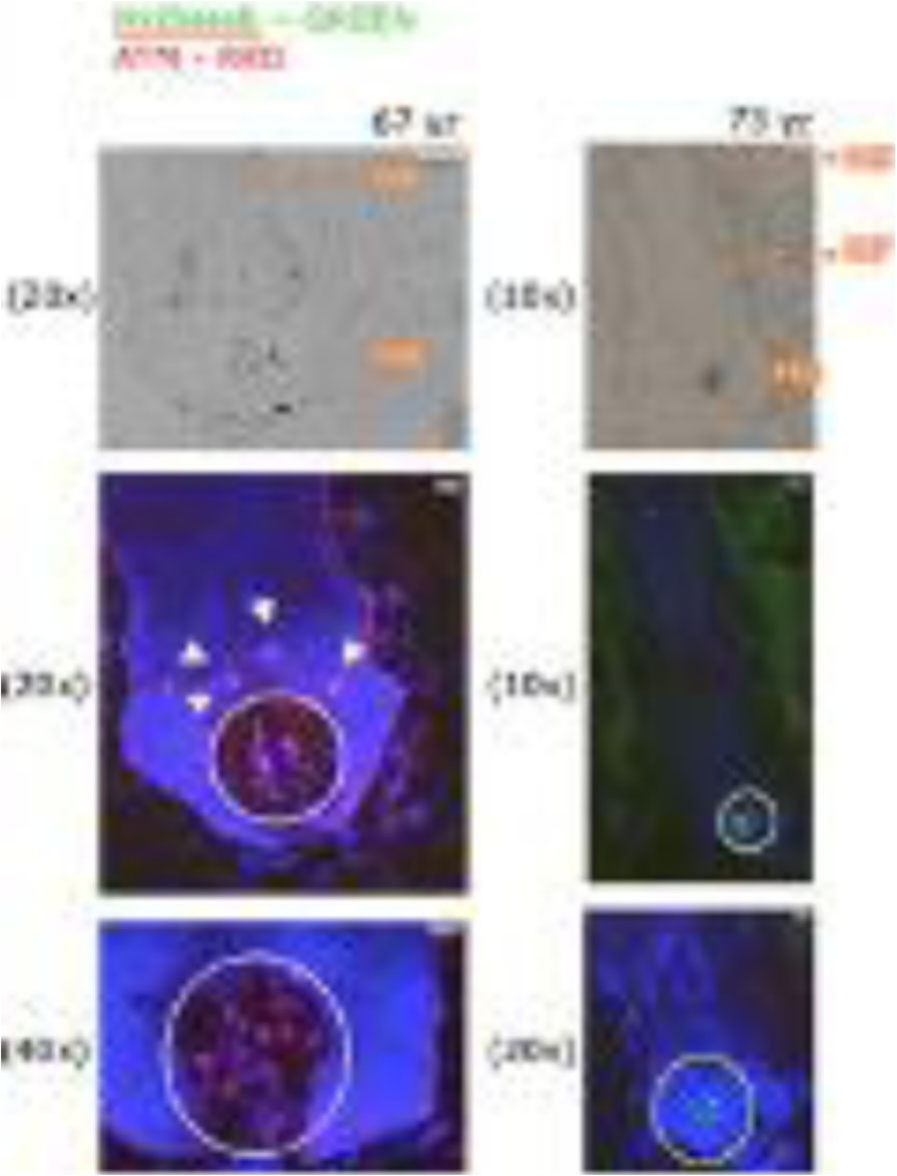
Expression of (NKibeteb+ATM). Melanocyte marker expression proportional to melanin observed in brightfield images (hair follicle(HF), hair shaft(HS), hair bulb(HB))

**Fig. 2 (abstract A16).**
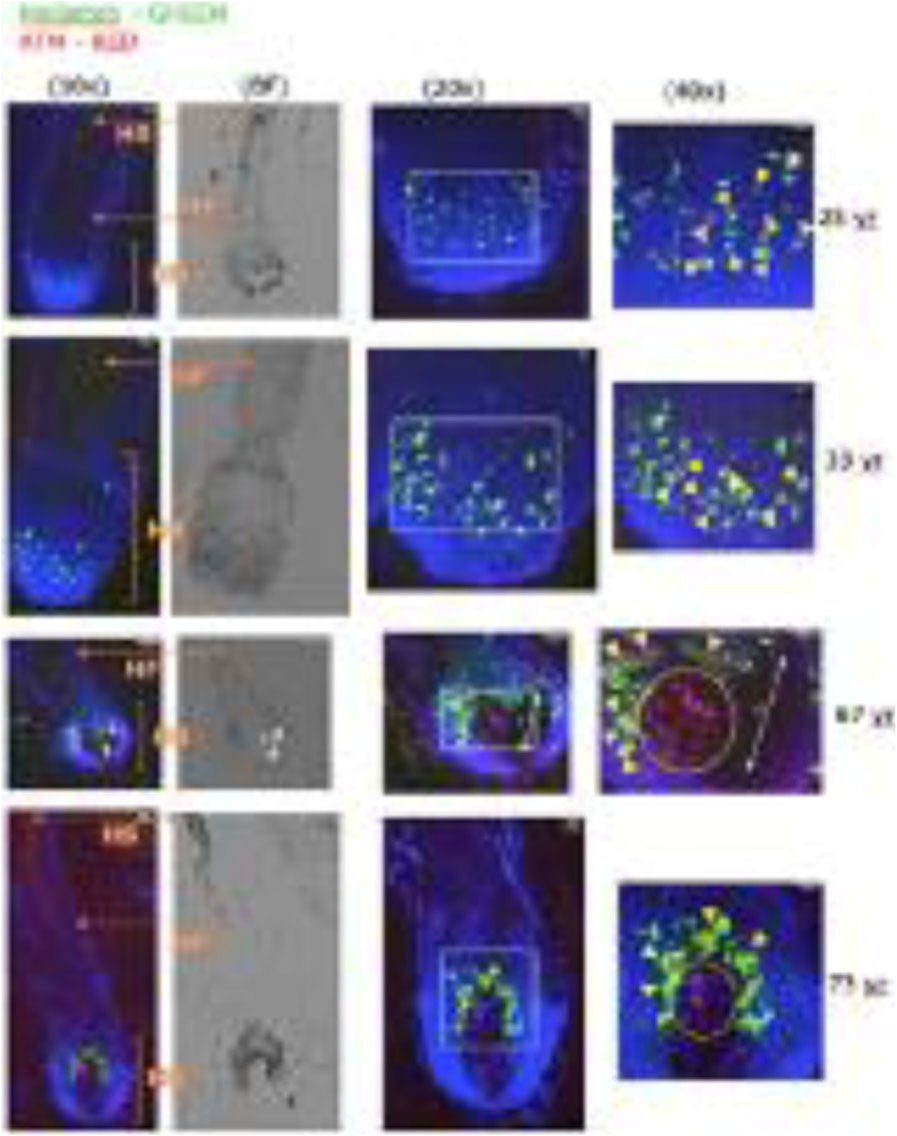
Expression of melanocyte marker (NKibeteb+ATM) in pigmented hair follicles with increasing age. (hair follicle(HF), hair shaft(HS), hair bulb(HB), triangles(melanocytes with ATM expression, tissue tear(T))

**Fig. 3 (abstract A16).**
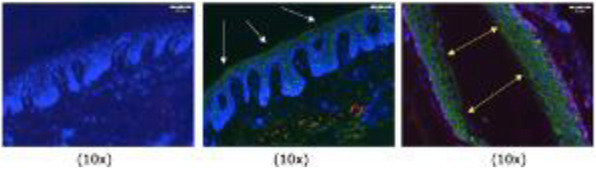
Expression of phospho-ATM(green) and ATM(red) in epidermis and upper HF outer root sheath

### A17: DUX4C overexpression dysregulates pathways implicated in facioscapulohumeral muscular dystrophy suggesting potential role in pathogenesis

#### Carter Bagley^1^, Darko Bosnakovski^2^, Micah D Gearhart^3^, Elizabeth T Ener^2^, Daniel Chi^2^, Michael Kyba^2^

##### ^1^UCD School of Medicine, University College Dublin, Belfield, Dublin, Ireland; ^2^Lillehei Heart Institute and Department of Pediatrics, University of Minnesota, Minneapolis, MN, USA; ^3^Department of Genetics, Cell Biology and Development, University of Minnesota, Minneapolis, MN, USA

###### **Correspondence:** Carter Bagley

**Background**

Facioscapulohumeral muscular dystrophy (FSHD) is a genetic neuromuscular disease caused by loss of repression of the *D4Z4* repeat array at 4q35. Each *D4Z4* repeat contains an open reading frame encoding DUX4, a transcription factor with a c-terminal transcriptional activation domain. *DUX4C* is a truncated and inverted *D4Z4* element encoding a protein identical to DUX4 except for its c-terminus, in which 82 amino acids are replaced by nonsense sequence. DUX4C expression inhibits myoblast differentiation and downregulates myogenic regulators *MyoD* and *Myf5*, suggesting a role in FSHD. We aim to identify the potential role of DUX4C in FSHD using bioinformatics analysis of mouse muscle with a muscle-specific doxycycline-inducible *DUX4C* gene.

**Materials and Methods**

RNA-seq data was obtained from mouse muscle after 2-week induction with doxycycline (controls lacked either the muscle-specific *rtTA* or the *iDUX4C* transgene). Differentially expressed genes (DEGs) were identified and analyzed in R using the Bioconductor suite.

**Results**

Overexpression of DUX4C dysregulates 3,513 genes (1,711 upregulated, 1,802 downregulated, padj<0.05). Comparison with 2-week DUX4 overexpression showed 474 DEGs shared, with 229 commonly upregulated, 202 commonly downregulated, and 43 oppositely regulated, suggesting DUX4C acts similar to DUX4 at the molecular level, causing a similar phenotype when overexpressed. 2,880 genes were uniquely affected by DUX4C, contributing to pathways involving Ca^2+^ homeostasis, oxidative stress, and apoptosis. DUX4C overexpression dysregulates the top 100 DUX4 early target genes (p=0.029), although expression changes were weak compared to DUX4. For example, the target *Myo1g* was induced to approximately 15% of the DUX4 fold-change.

**Conclusions**

Despite lacking the transcriptional activation domain DUX4C causes numerous transcriptional changes similar to DUX4 and produces a similar muscle pathology in mice. DUX4C overexpression induces some direct targets of DUX4, but not as strongly suggesting it has much weaker transcriptional activity. This data coupled with DUX4C’s dysregulation of known FSHD pathways gives cause for further research to be conducted to determine the extent of its action and its significance in FSHD in humans.

### A18: Muscle wasting in a breast cancer model: changes in muscle fibre size and mitochondrial dynamics

#### Jacob Lavieille-Curran^1^, Róisín Dwyer^2^, Katarzyna Whysall^3,4^

##### ^1^School of Medicine, National University of Ireland Galway, Galway City, Galway, Ireland; ^2^Lambe Institute for Translational Research, National University of Ireland Galway, Galway City, Galway, Ireland; ^3^Department of Physiology, School of Medicine, National University of Ireland Galway, Galway City, Galway, Ireland; ^4^Institute of Ageing and Chronic Disease, University of Liverpool, Liverpool, Merseyside, United Kingdom

###### **Correspondence:** Jacob Lavieille-Curran

**Background**

Cancer cachexia is a severe muscle wasting condition, affecting up to 50% of cancer patients [1]. It is linked to reduced treatment tolerance and response, and increased morbidity and mortality [2]. As the exact mechanism of cancer cachexia is not understood, further investigations are crucial to finding an effective therapy. Mitochondria are greatly affected in catabolic conditions of muscle loss [3] and dysfunctional mitochondria can trigger catabolic signalling pathways causing muscle atrophy [3]. This project aims to determine changes in muscle in a mouse model of breast cancer and investigate potential associated mechanisms.

**Materials and Methods**:

The gastrocnemius and tibialis anterior were isolated from 3 control mice and 3 tumour-bearing mice. The muscles were cryosectioned at 8μm and fluorescently imaged at 20x magnification (Fig 1). RNA was isolated and used for quantitative polymerase chain reaction to establish changes in the expression of mitochondrial and atrophy-related genes.

**Results:**

No significant difference was found between muscle fibre sizes in control mice and mice with cancer (Fig 2). Significant decrease in the relative expression of the mitochondrial genes Tomm20 and MT-ND1 was observed in tibialis anterior samples from tumour-bearing mice. Significant decrease in the relative expression of the mitochondrial genes COXIV and OPA1 was also observed in gastrocnemius samples from tumour-bearing mice.

**Conclusion**

The weight of tibialis anterior, but not gastrocnemius muscles, was significantly reduced in tumour-bearing mice compared to control mice. However despite this and contrary to findings from human studies [2], muscle fibre diameter and distribution did not show a significant difference between tumour-bearing mice and control mice, potentially due to detection challenges of the atrophic muscle fibres. Further investigations using larger sample sizes and different time points from tumour induction are necessary. Expression of Tomm20 and MT-ND1 was reduced in the tibialis anterior muscle of tumour-bearing mice, while the expression of COXIV and OPA1 was reduced in the gastrocnemius muscle of tumour-bearing mice. These changes may indicate cancer cachexia’s effect on biogenesis of mitochondria (Tomm20), the oxidative phosphorylation process (COXIV and MT-ND1), cellular atrophy and mitochondrial stability (OPA1) within skeletal muscle and may be linked to fibre type-specific changes in these processes.

**Acknowledgements**

This project was funded by the National Breast Cancer Research Institute, and was made possible with the help of Dr. Katarzyna Goljanek-Whysall and the Golajnek-Whysall Lab and Dr. Róisín Dwyer.

**References**

1. Winje IM, Sheng X, Hansson KA, Solbrå A, Tennøe S, Saatcioglu F, Bruusgaard JC, Gundersen K. Cachexia does not induce loss of myonuclei or muscle fibres during xenografted prostate cancer in mice. Acta Physiol. 2019; 225(3).

2. Johns N, Hatakeyama S, Stephens NA, Degen M, Degen S, Frieauff W, Lambert C, Ross JA, Roubenoff R, Glass DJ, Jacobi C, Fearon KC. Clinical classification of cancer cachexia: phenotypic correlates in human skeletal muscle. PLoS One. 2014; 9(1).

3. Romanello V, Sandri M. Mitochondrial Quality Control and Muscle Mass Maintenance. Front Physiol. 2016; 6:422.

**Fig. 1 (abstract A18).**
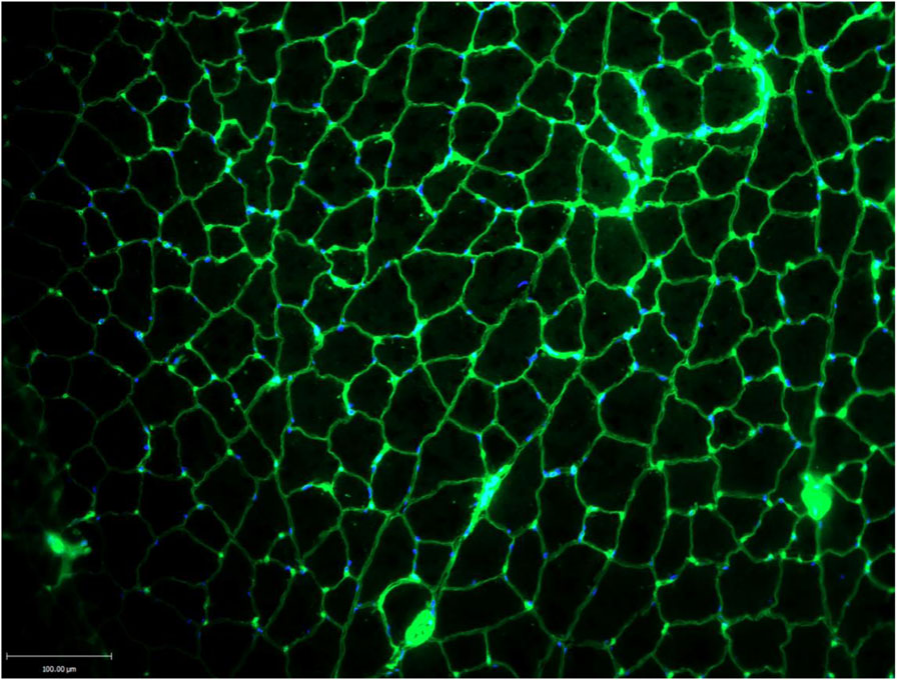
Mouse muscle fibre under fluorescent microscopy

**Fig. 2 (abstract A18).**
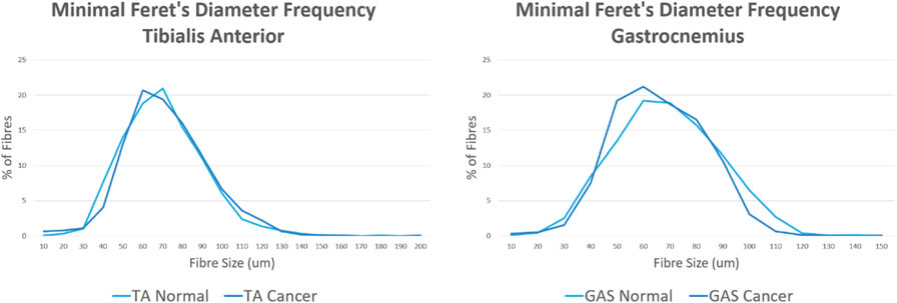
Frequency distribution of muscle fibre size in tumour-bearing mice

### A19: Investigating macrophage activation in response to damage-associated molecular patterns in multiple sclerosis

#### Devika Dahiya^1^, Caitlyn Joy Loo^1^, Jennifer Dowling^2^, Claire McCoy^2^

##### ^1^School of Medicine, Faculty of Medicine and Health Sciences, Royal College of Surgeons in Ireland, 123 St. Stephen's Green, D02 YN77 Dublin, Ireland; ^2^School of Pharmacy and Biomolecular Sciences, Royal College of Surgeons in Ireland, 123 St. Stephen's Green, D02 YN77 Dublin, Ireland

###### **Correspondence:** Devika Dahiya

**Background**

Although the cause of Multiple Sclerosis (MS) is unknown, we understand that active macrophages release inflammatory mediators causing symptomatic damage. However, the trigger for macrophage activation is unclear. We investigated if the Damage Associated Molecular Pattern, High-mobility-group-box 1 (HMGB1), can trigger macrophage activation. HMGB1 is a ubiquitous nuclear architectural protein and was found to be upregulated in CSF samples and active plaques of MS patients [1].

**Materials and methods**

We measured the hallmarks of macrophage activation; NO (Greiss assay), IL-6, TNFα

and IL-1β (ELISA), and miR-155 (RT-PCR). Raw 264.7 and bone marrow-derived macrophages were stimulated in a dose and time-dependent manner with HMGB1 and Toll-like receptor agonist, LPS as a positive control. All experiments were performed in triplicate, 4 independent times.

**Results**

At 4, 6, 24 and 48 hrs, LPS (1 mg/ml) induced activation markers (NO, IL-6, TNFα

and IL-1β) in a time-dependent manner in both cell lines as expected. This was greatest with LPS at 48 hrs. HMGB1 alone (5, 10 and 50 ng/ml) did not have any impact on any of the activation parameters. However, when stimulated with both LPS and HMGB1, for 48 hrs, a statistically significant synergistic effect in NO, IL-6, TNFα

and IL-1β production by Raw264.7 was seen.

**Conclusions**

Our investigations imply that HMGB1 can synergistically enhance an inflammatory response in LPS stimulated macrophages. The impact of HMGB1 at later time points suggests it may worsen chronic inflammation, which may be relevant in MS patients who have increased HMGB1 and suffer chronic inflammation. Further characterisation will explore the different domains of the HMGB1 molecule on macrophage activation, as well as the mechanism of enhanced macrophage activation markers.

**Acknowledgements**

Research PI: Dr Claire McCoy

Research Supervisor: Dr Jennifer Dowling

Research Department: School of Pharmacy and Biomolecular Sciences

Research Partner: Caitlyn Joy Loo

RCSI Research Summer School: Dr Sarah O’Neill

Funding: The Wellcome Trust

**Reference**

1. Andersson, Å., Covacu, R., Sunnemark, D., Danilov, A.I., Dal Bianco, A., Khademi, M., Wallström, E., Lobell, A., Brundin, L., Lassmann, H. and Harris, R.A. Pivotal Advance: HMGB1 expression in active lesions of human and experimental multiple sclerosis. Journal of Leukocyte Biology. 2008; 84: 1248-1255. 10.1189/jlb.1207844

### A20: Systematic review on the clinical presentation and management of the COVID-19 associated multisystem inflammatory syndrome in children (MIS-C)

#### Marah Shaikh Yousef, Nur Syazana Idris, Charles Yap, Abdulaziz Abdullah Alsubaie, Pramath Kakodkar

##### School of Medicine, National University of Ireland Galway, Galway City, Republic of Ireland

###### **Correspondence:** Pramath Kakodkar

**Background:**

Firstly, we collated the vast repository of MIS-C cases and presented them in a simplified, condensed, and comprehensive format. Secondly, we explored the clinical presentation, and efficacy of the management options. Additionally, we briefly discussed the pathophysiology and addressed the variance in the jargon and criteria relating to this condition.

**Methods:**

A systematic literature review was conducted on the 17^th^ of October 2020 in accordance with PRISMA (2015) guidelines. The search terms: ‘MIS-C’, ‘Kawasaki-like Disease’, ‘PIMS-TS’, and ‘COVID-19’ were queried on Medline and Embase databases. Publications that fulfilled the inclusion criteria were included and were assessed for parameters pertaining to the clinical course and management.

**Results:**

From December 2019 to October 2020, 131 publications were identified. Of these, 56 publications (n=646 patients) fit the inclusion criteria. Median age was 10 years (range: 0.5-17 years), 52.2% (n=337/646) were male, and 33.5% (n=128/382) were of African ethnicity. SARS-CoV-2 reverse transcriptase PCR and serology were positive in 42% (n=142/426), and 85.3% (n=300/352) of cases respectively. Presenting complaint(s) were predominately gastrointestinal: 77.6% (n=436/562) generalized abdominal pain, 76.4% (n=386/505) vomiting, and 63.2% (n=203/321) diarrhea. Hypotensive shock was also commonly observed at admission. Additionally, laboratory data revealed elevated neutrophils and inflammatory markers. Echocardiogram findings indicated reduced left ventricular ejection fraction and myocarditis in 22.6% (n=85/376) and 22.3% (n=84/376) of cases, respectively. Immunoglobulins and intravenous steroids were predominantly used in 76% (n=433/571) and 51% (n=317/618) of cases, respectively. Majority of the patients (97%, n=418/431) were discharged home. A combination treatment of Tocilizumab and IVIG had a mean length of stay of 7±3 days and 95.5% (n=21/22) discharge rate with low complications in comparison to either of the treatments alone.

**Conclusion:**

MIC-S syndrome is a pediatric hyperinflammatory condition that has an association with COVID-19 background exposure. MIS-C has a heterogeneous multisystem presentation that can be associated with life threatening cardiac complications. There is a need to further explore its long-term morbidity.

### A21: An audit on the use of brain biopsies for non-neoplastic brain diseases

#### Derry O’Flynn^1^, Dr Niamh Bermingham^2^

##### ^1^School of Medicine, University College Cork, Cork, Ireland; ^2^Department of Neuropathology, Cork University Hospital, Cork, Ireland

###### **Correspondence:** Derry O’Flynn

**Background**

In cases where a brain tumour is seen on neuroimaging brain biopsies are the gold standard method for confirming the diagnosis. Brain biopsies have a less predictable role in the investigation of patients with a neurologic syndrome of unknown aetiology in whom a neoplastic process is not suspected and are not frequently performed in this setting. Brain biopsy tend to be the last diagnostic resource in these disorders and the value of this procedure on diagnosis as well as outcome/management is less well-defined. The aim of this study was to evaluate the diagnostic yield and safety of brain biopsies performed in a single Neurosciences centre for clinically non-neoplastic brain diseases; to compare the pathologic evaluation with pre-biopsy diagnosis; and to analyse if biopsy results significantly altered patient management.

**Materials and methods**

This is a retrospective study of brain biopsies for non-neoplastic brain diseases performed at a single Neurosciences centre over a period of 10 years. Cases were identified using neuropathology reports and cases were excluded if the pre-operative diagnosis was clearly malignancy. The relationship between preoperative presenting complaint, laboratory testing, neuroimaging and management were examined and compared with post-biopsy clinical outcome as well as management changes.

**Results**

Twenty cases were identified from pathology reports. Sixteen of the twenty biopsies (80%) were pathologically diagnostic. Amyloid angiopathy was the most common diagnosis (41.7%) while demyelinating disorder and vasculopathy each accounted for 16.7% of the diagnoses made. Following biopsy results, clinical management altered in 80% of patients with the introduction of immunosuppressive agents being the most common change (occurring in 43.8%). 15.4% of patients with clinical outcome recorded returned to pre-disease baseline and a further 46.2% of patients with a recorded clinical outcome improved without returning to pre-disease baseline. Complications were rare but did not impact overall outcome.

**Conclusions**

This study shows that brain biopsies, when appropriately performed, are useful and have a high diagnostic yield even independently of other investigative modalities. Clinical outcome was, for the majority, positive with a large proportion of the patients improving clinically. Complications were rare and did not impact the patients overall outcome. This shows that brain biopsies are useful and safe as part of the diagnostic algorithm for non-neoplastic neurological conditions of uncertain or ambiguous aetiology and impact significantly on patient management.

### A22: Significance of placental swab in diagnosing vertical transmission in SARS-CoV-2 positive mothers

#### Isabelle Sweeney^1^, Al Assaf Niazy^2^, Khan Rizwan^2^

##### ^1^Graduate Entry Medical School, University of Limerick, Limerick, Ireland; ^2^Department of Neonatology, University Maternity Hospital Limerick, Limerick, Ireland

###### **Correspondence:** Isabelle Sweeney (sweeneyi@tcd.ie)

**Background**

Currently, there is limited date on the effects of COVID-19 on pregnancy and neonatal outcome. This literature review aims to investigate the possibility of fetal vertical transmission in COVID-19 positive pregnant mothers by diagnosing through placental swabs.

**Materials and methods**

The search terms ‘pregnant COVID-19 positive mothers’, ‘fetal vertical transmission’ and ‘placental swabs’ were used. 11 papers were selected for this review.

**Results**

This literature review comprises 45 COVID-19 positive pregnant women whose placentas and neonates were also analysed by RT-PCR for the presence of SARS-CoV-2. 43 neonates were successfully delivered primarily via caesarean section out of 45 expectant mothers (96%). 2 mothers did not deliver due to severe preeclampsia and a miscarriage both occurring in the second trimester. 3 neonates tested positive for SARS-CoV-2 (7%). We report no neonatal mortality after birth and no maternal mortality. 8 female’s placentas tested positive for SARS-CoV-2 out of a total of 45 tested (18%). Of these 8, 2 cases of SARS-CoV-2 were identified in the maternal, neonatal and placental tissue.

**Conclusions**

After reviewing multiple studies and investigating the nature of placental physiology in SARS-CoV-2 positive mothers we conclude that there is no concrete evidence of vertical transmission occurring between mother and infant. However, there are inconsistencies across the different papers used for this review and further research investigating the effects of COVID-19 on pregnant women by using RT-PCR to test the mother, placenta, vaginal fluid, breast milk and infant for SARS-CoV-2 at various stages of transmission is urgently needed.

### A23: Pain detectives: Optimizing the management of pain and irritability in children with severe neurological impairment

#### Ketchum K^1,11^, Hermansen AM^1^, Andrews G^1^, Pawliuk C^1^, Dewan T^9^, Gnanakumar V^10^, Orkin J^4,5^, Richardson A^1^ Vadeboncoeur C^6,7,8^, Holsti L^1,3^, Carleton B^1,2^, Oberlander T^1,2^, Siden H^1,2^

##### ^1^British Columbia Children’s Hospital Research Institute, Vancouver, Canada; ^2^Department of Pediatrics, University of British Columbia, Vancouver, Canada; ^3^Department of Occupational Science and Occupational Therapy, University of British Columbia, Vancouver, Canada; ^4^Department of Pediatrics, University of Toronto, Toronto, Canada; ^5^Complex Care, Hospital for Sick Children, Toronto, Canada; ^6^Department of Pediatrics, University of Ottawa, Ottawa, Canada; ^7^Children’s Hospital of Eastern Ontario, Ottawa, Canada; ^8^Roger Neilson House, Ottawa, Canada; ^9^Department of Pediatrics, University of Calgary, Calgary, Canada; ^10^Physical Medicine and Rehabilitation, Alberta Children’s Hospital, Calgary, Canada; ^11^Medical student, University College Dublin, Dublin, Ireland

###### **Correspondence:** Ketchum K (katherine.ketchum@ucdconnect.ie)

**Background**

Children with severe neurological impairment (SNI) are amongst the most vulnerable patients seen by clinicians. Children with SNI are typically non-verbal, non-mobile and cognitively impaired [1]. Many of these children experience pain or demonstrate irritability on a daily basis [2]. The source of this discomfort is difficult to identify when these children are unable to communicate. Due to the complex nature of these sensations, we use the term Pain and Irritability of Unknown Origin (PIUO) to describe them. We believe that lack of a standardized approach to investigating and managing PIUO may be contributing to pain persistence and suffering in this population. To address this, our team developed a systematic approach for the assessment of pain in patients with SNI. The PIUO Pathway, as we have termed it, incorporates structured history-taking, physical examination, screening investigations for known and occult sources of pain, and pharmacological interventions. The goal is to efficiently assess treatable causes of nociceptive-inflammatory pain.

**Methods**

We used a waitlist-controlled randomized trial to test the efficacy of the PIUO Pathway. Participants were randomized to PIUO Pathway or waitlist (standard care) arms. The Pathway was deemed a success if the source of nociceptive-inflammatory pain was identified or if a participant was pain-free/had low pain on two consecutive visits. Secondary outcomes to be studied included decreased pain severity, improved family quality of life and ease of implementation of the PIUO Pathway for clinicians.

**Results**

This trial is ongoing. Preliminary results show that 23 children have completed the PIUO Pathway. Four participants have had their source of pain identified, and treatment strategies for these children are currently being investigated. Seven other participants have had their PIUO identified and resolved. Recruiting will continue across our four Canadian research sites until 120 participants have completed the Pathway. Results for secondary outcomes will be assessed upon trial completion. Children in whom a source of pain remains unidentified or who continue to have pain after completion of the PIUO Pathway will be eligible to participate in our next trial, which is a prospective N-of-1 randomized controlled trial which will investigate the efficacy of gabapentin in relieving PIUO in children with SNI.

**Acknowledgments**

The authors would like to thank our participants and their families for joining our study. This research is funded by the CHILD-BRIGHT Network, SPOR and CIHR.

**Trial Registration Number**

ClinicalTrials.gov Identifier: NCT03464773 (PIUO Pathway trial)

ClinicalTrials.gov Identifier: NCT04619862 (gabapentin trial)

**References**

1. Siden HB, Carleton BC, Oberlander TF. Physician variability in treating pain and irritability of unknown origin in children with severe neurological impairment. Pain Res Manag J Can Pain Soc J Société Can Pour Trait Douleur. 2013;18(5):243-248.

2. Ståhle-Öberg L, Fjellman-Wiklund A. Parents’ experience of pain in children with cerebral palsy and multiple disabilities – An interview study. Adv Physiother. 2009;11(3):137-144. doi:10.1080/14038190902906318.

### A24: Pathway analysis of splice variants suggest a role for inflammatory processes in type 1 myotonic dystrophy

#### Iulia Cornila, Jeffrey C. Glennon

##### Conway Institute of Biomolecular and Biomedical Research, UCD School of Medicine, Belfield, Dublin 4, Ireland

###### **Correspondence:** Iulia Cornila

**Background**

Type 1 myotonic dystrophy (DM1) is a multi-system neuromuscular disease caused by a trinucleotide expansion of the *DMPK* gene, which results in toxic gain-of-function activity of RNA, leading to a myriad of downstream splice variants and aberrant proteins. This research will explore if the splice variants share any common functionalities or pathway interactions.

**Materials and methods**

From existing published literature, experimentally validated and predicted splice variants in DM1 were analyzed using pathway analysis to determine if they contribute to common biological pathways. In total, 83 predicted and 55 experimentally validated splice variants in DM1 were examined using KEGG mapper and Reactome databases, with statistics computed using Reactome over-representation analysis. MicroRNA predicted to regulate misspliced genes were gathered using TargetScan:Human, and their pathway analysis was also conducted in order to search for common functionality between the two datasets.

**Results**

Predicted misspliced DM1 genes revealed strong pathway interactions in responses of the innate and adaptive immune system: antigen processing/presentation (p<1.11E-16), endosome and phagosome pathways (p<1.11E-16), and cytokine signalling (p<2.74E-14). Experimentally validated misspliced DM1 genes showed significant pathway interactions involving: MECP2 regulation of gene transcription (p<1.7E-7), cardiac conduction (p<1.57E-4), RTK signal transduction (p<2.26E-3), platelet homeostasis (p<2.53E-3), ion channel transport (p<2.79E-3) and integrin signalling (p<3.99E-3). Both gene sets implicated pathways involved in muscle contraction (p<5.05E-4) and myogenesis (p<0.0341). MicroRNA analyses revealed significant pathway interactions in the immune system, platelet homeostasis, myogenesis and muscle contraction, axon guidance, and intracellular signal transduction.

**Conclusions**

Pathway analysis of DM1 variant genes and their regulatory microRNA shows common functionality pathways prominently in immune system operations (e.g. interleukin and interferon signalling, and lymphocyte activity). These are especially present in the predicted misspliced gene set which has yet to be experimentally explored. Future studies should confirm which of these common pathways could be viable mechanisms for intervention or biomarkers of prognosis/ disease severity.

### A25: Lower incidence of preterm birth in Irish cohort with congenital uterine anomalies

#### Lily Farrell^1^, Niamh Keating^2^, Siobhán Corcoran^2^

##### ^1^University College Dublin, Dublin, Ireland; ^2^Pre-Term Birth Clinic, National Maternity Hospital, Ireland

###### **Correspondence:** Lily Farrell

**Background**

Globally, congenital uterine anomalies have a strong association with an increased risk of adverse pregnancy outcomes including miscarriage (43%) [1], pre-term birth (>20%) [2] and caesarean delivery (>34%) [2]. The objective of this study is to examine the obstetric outcomes for women with significant uterine anomalies in the National Maternity Hospital, Ireland (NMH).

**Material and methods**

This is a retrospective cohort study of all cases of uterine anomalies in pregnancy in the NMH over a 10 year period (2011-Present). Cases were identified by reviewing the database of the NMH’s Preterm Birth Clinic, where all women with known uterine anomalies are referred for their antenatal care. Within this time frame, there have been 33 such pregnancies amongst 19 women. A significant uterine anomaly was defined as Unicornuate, Bicornuate, Didelphys and Septate Uterus requiring resection, with or without a Vaginal Septum.

**Results**

Within this study period (2011-2020), the NMH reported 86,535 deliveries amongst 94,141 women. Of these women, 19 (0.00024%) were identified as having a significant uterine anomaly. These ranged from Bicornuate Uterus (n=9; 47.4%), Uterine Didelphys (n=5; 26.3%), Resected Uterine Septum (n=2; 10.5%), Uterine Septum (n=1; 5.3%), Unicornuate Uterus (n=1; 5.3%) and Vaginal Septum (n=4; 21.1%). There were no cerclages. Under Pre-Term surveillance (averaging 11 visits per pregnancy), there were 32 livebirths (Table 1). One pregnancy remains on-going.

**Conclusions**

Our study found a notably lower preterm birth rate (3.1%) amongst this cohort of women relative to the findings of International studies where rates as high as 39.7% were recorded. Our miscarriage rate (13.5%) was also lower than comparable studies (42.9%). The caesarean delivery rate was higher amongst this cohort relative to the background population and international findings. However, this was largely attributed to elective sections due to increased maternal age and patient demand. Insights gained from this data will help us to focus counselling in our own specific population regarding the pregnancy outcomes of women with uterine anomalies. Our findings suggest that this area warrants further national investigation. We recommend a similar audit be carried out in the remaining Irish Maternity Hospitals using the same methodology. This could prove invaluable if nationwide results differ significantly from ours in which case key differentiating factors may be identified.

**References**

1. Jayaprakasan K, Chan YY, Sur S, Deb S, Clewes JS, Raine-Fenning NJ. Prevalence of uterine anomalies and their impact on early pregnancy in women conceiving after assisted reproduction treatment. Ultrasound Obstet Gynecol. 2011 Jun;37(6):727-32. doi: 10.1002/uog.8968. Epub 2011 Mar 30. PMID: 21337662.

2. Meiling Hua, Anthony O. Odibo, Ryan E. Longman, George A. Macones, Kimberly A. Roehl, Alison G. Cahill, Congenital uterine anomalies and adverse pregnancy outcomes, American Journal of Obstetrics and Gynecology, Volume 205, Issue 6, 2011, Pages 558.e1-558.e5, ISSN 0002-9378, https://doi.org/10.1016/j.ajog.2011.07.022.

**Table 1:** **(abstract A25).** Comparison of Our Study Population versus General Hospital Obstetric Outcomes

Variable	Study Population (n=32)	Background population (n=79,832)*
**Average gestation at delivery**	39+4	37+0 to 41+6
**Range of GA (Gestational Age)**	[33+4 to 42+0]	[24/40 to 42/40]
**Mode of Delivery - Caesarean Section**	14 (43.8%)	20,205 (25.3%)
**% Elective CS**	10 (31.3%)	
**% Emergency CS**	4 (12.5%)	
**PTB < 34+0**	1 (3.1%)	1994 (2.5%)
**PTB 24+0 – 37+0**	1 (3.1%)	5391 (6.76%)
**% Miscarriages**	13.5% (n=5)	
**% of First Trimester Miscarriages**	10.8% (n=4)	
**% of Second Trimester Miscarriages**	2.7% (n=1)	
**IUGR**	0%	

*NMH annual clinical reports 2011-2019

### A26: Frailty in hospitalized patients: Upholding a standard of care

#### Anna M Demian^1^, Katherine Coupland^1^, Ming-Chih Tsai^2^, Joseph Garvin^2^, Niamh Hannon^3^

##### ^1^School of Medicine, University of Limerick, Limerick, Ireland; ^2^Department of General Surgery, Portiuncula University Hospital, Ballinasloe, Ireland; ^3^Department of Geriatric Medicine, Galway University Hospital, Galway, Ireland

###### **Correspondence:** Anna M Demian

**Background**

The geriatric population is defined as those over the age of 65 years [1]. This patient population comprises >14.15% of Ireland’s total population and 53% of inpatient hospital care [2, 3]. Thus, it is important for health care professionals (HCP) to identify those that are frail and in need of comprehensive geriatric care. This audit sought to examine Portiuncula University Hospital’s (PUH) compliance with the Health Service Executive (HSE) guidelines which states that all older adult patients identified as being frail or at risk of frailty should have a comprehensive geriatric assessment (CGA) [1].

**Materials and Methods**

To assess the congruence of PUH and the HSE guidelines, a questionnaire was distributed to 131 PUH HCP evaluating the level of awareness of frailty and confidence in managing frail patients. Additionally, 59 geriatric surgical patient charts were reviewed to assess PUH adherence with the use of standardized frailty tools and appropriate referrals for geriatric surgical patients admitted to hospital. The next steps will include educating HCP and re-auditing 3 months after.

**Results**

Results from the questionnaire showed discrepancy between confidence reported (32%) and factual knowledge (68%) of frailty assessment amongst PUH HCP (Figure 1 and Figure 2). Results also showed that all hospital departments would benefit from formal education. Lastly, based on the guidelines PUH is underutilizing frailty assessment tools and referrals for CGA’s with only 17% of all geriatric surgical inpatients being appropriately referred, 2% having a Rockwood score and 4% having a 4AT score completed.

**Conclusion**

The results of this audit will help target future research and tailor educational interventions. It is evident from the results that education is needed for all HCP at PUH in order to maximize utilization of standardized frailty assessments. Future goals are to establish a database on geriatric care in PUH for research purposes and expand the database to include other departments.

**References**

1. National Clinical Programme for Older People. Comprehensive Geriatric Assessment: a summary. Health Service Executive. 2016; 1:1-6. Available at https://www.hse.ie/eng/services/publications/clinical-strategy-and-programmes/comprehensive-geriatric-assessment-summary.pdf. Accessed 26 June 2020.

2. Central Statistics Office. Census of population 2016: profile 3 an age profile of Ireland. CSO statistical publication. 2017. Available at https://www.cso.ie/en/releasesandpublications/ep/p-cp3oy/cp3/. Accessed 24 July 2020.

3. Department of Health. Health in Ireland: key trends 2018. Statistics and Analytics Unit. 2019; 36-47. Available at health.gov.ie. Accessed 24 July 2020.

**Fig. 1 (abstract A26).**
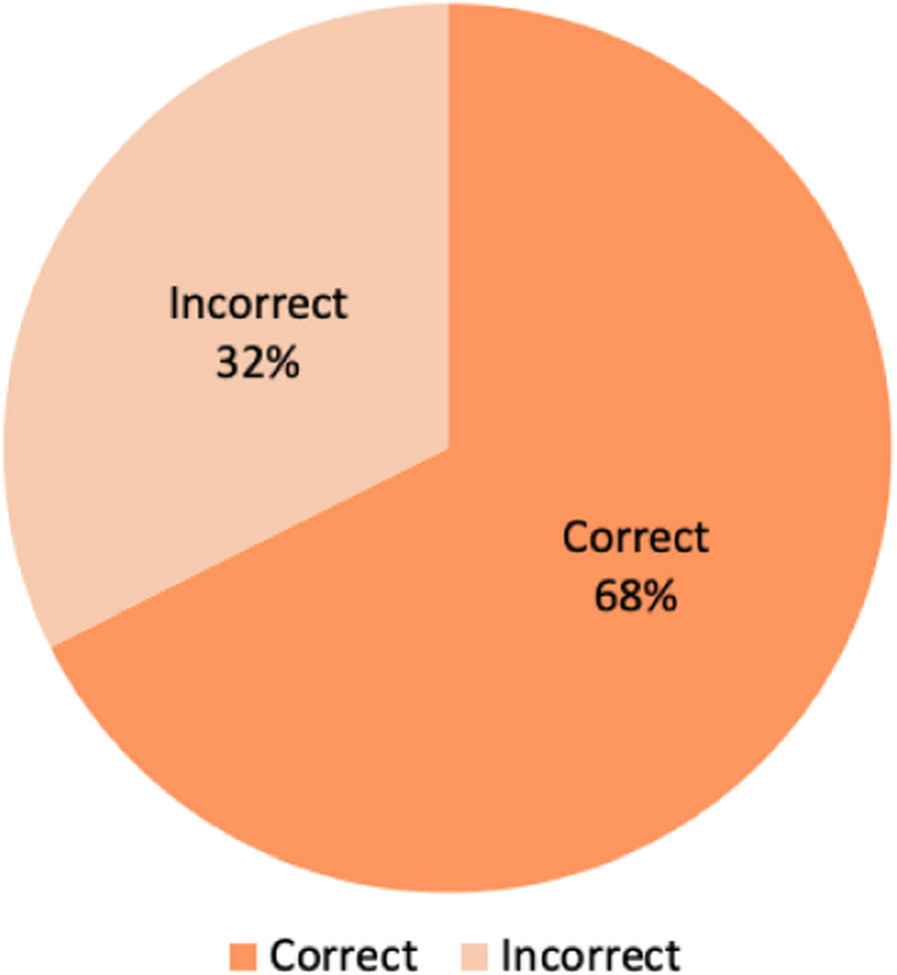
Average correct and incorrect answers amongst all PUH HCP (%) (n = 131)

**Fig. 2 (abstract A26).**
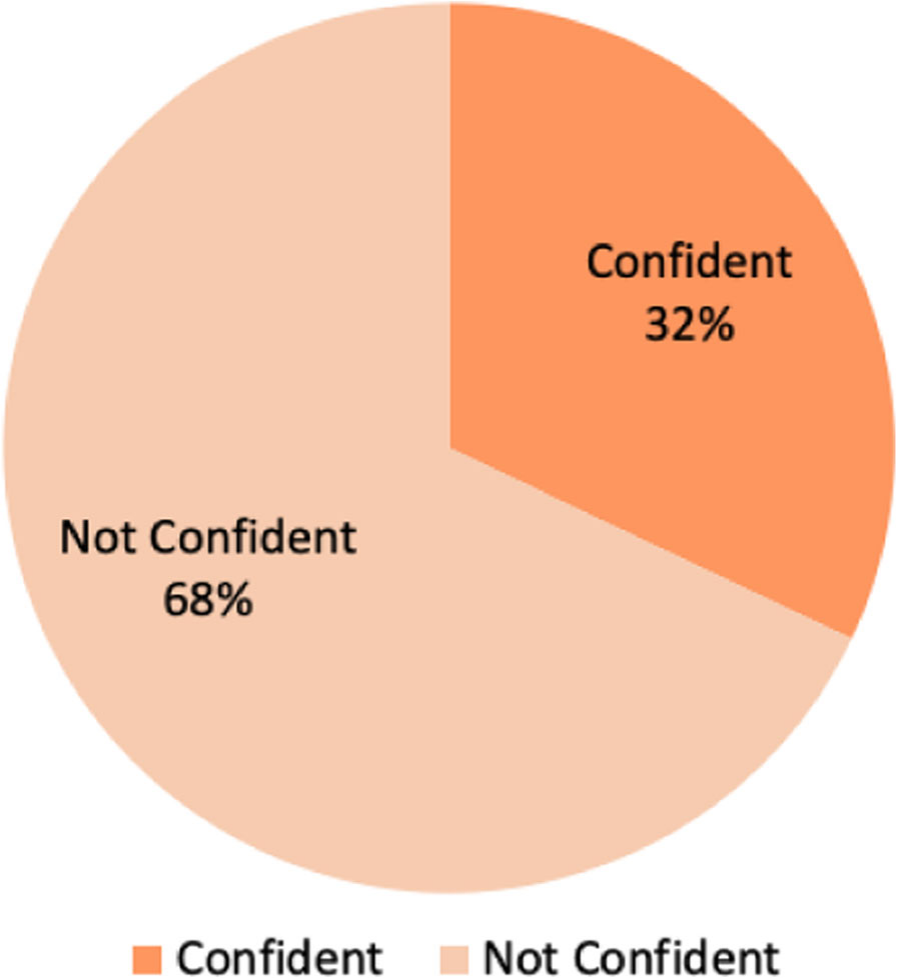
Determining the confidence of PUH HCP in assessing frailty (%) (n= 131)

### A27: Appropriate non-vitamin K antagonist oral anticoagulant dosing in non-valvular atrial fibrillation patients

#### Debola Shomoye (debolasho@yahoo.ie)

##### School of Medicine, University of Limerick, Garraun, Castletroy, Limerick, Ireland

**Background**

Atrial fibrillation (AF) is the most common clinical arrhythmia, carrying an approximate lifetime risk of one in four after age 40 years [1]. Only anticoagulation has been proven to reduce AF-related mortality [2]. Inappropriate NOAC doses lead to less favourable outcomes in relation to thromboembolic events and death [3]. This project aims to determine the degree of compliance with Non-Vitamin K Antagonist Oral Anticoagulant (NOAC) dosing standards and to implement a computerised NOAC dosing aid to reduce the occurrence of inappropriate NOAC dosing in non-valvular atrial fibrillation (NVAF) patients.

**Materials and Methods**

Data from 100 living NVAF patients on NOACs was captured by a CompleteGP (CGP) software search which utilised Boolean operators. Variables of interest were patient age, NOAC dosage and frequency of renal function and body weight testing. Appropriate dosing was calculated based on information in the approved European Union Summary of Product Characteristics (SPC).

**Results**

13% patients were on an incorrect NOAC dose (Figures 1&2). 14% patients did not have baseline biochemical or weight results prior to initiation of NOAC therapy. Moreover, 44% had not undergone regular testing since commencing NOAC therapy. Of the total number of patients in each drug cohort eligible for standard doses, 89% apixaban, 88% rivaroxaban, 100% edoxaban and 100% dabigatran patients appropriately received standard doses. Underdosing was the only form of inappropriate dosing in patients on rivaroxaban (n=3). 18% patients eligible to receive the standard daily dose of apixaban were prescribed a reduced dose (n=8). This represented 73% of all apixaban inappropriate dosing. Meanwhile, 12.5% of all rivaroxaban patients eligible for a standard dose were prescribed a reduced dose. A computerised dosing aid was designed to improve compliance with dosing recommendations and prompt prescribers to arrange renal function and bodyweight testing reminders for NVAF patients (Figures 3, 4).

**Conclusion**

Inappropriate NOAC dosing is prevalent in general practice and detrimental to patient health. Compliance with NOAC dosing standards in AF patients can be optimised by utilising a computerised dosing aid and formal reminder systems.

**References**

1. Lloyd-Jones DM, Wang TJ, Leip EP, Larson MG, Levy D, Vasan RS, D’Agostino RB, Massaro JM, Beiser A, Wolf PA, Benjamin EJ. Lifetime risk for development of atrial fibrillation: the Framingham Heart Study. Circulation. 2004; 110:1042-6.

2. Wolf PA, Abbott RD, Kannel WB. Atrial fibrillation as an independent risk factor for stroke: the Framingham Study. Stroke. 1991; 22:983-8.

3. Steinberg BA, Shrader P, Pieper K, Thomas L, Allen LA, Ansell J, Chan PS, Ezekowitz MD, Fonarow GC, Freeman JV, Gersh BJ. Frequency and Outcomes of Reduced Dose Non–Vitamin K Antagonist Anticoagulants: Results From ORBIT‐AF II (The Outcomes Registry for Better Informed Treatment of Atrial Fibrillation II). Journal of the American Heart Association. 2018; 7:e007633.

**Fig. 1 (abstract A27).**
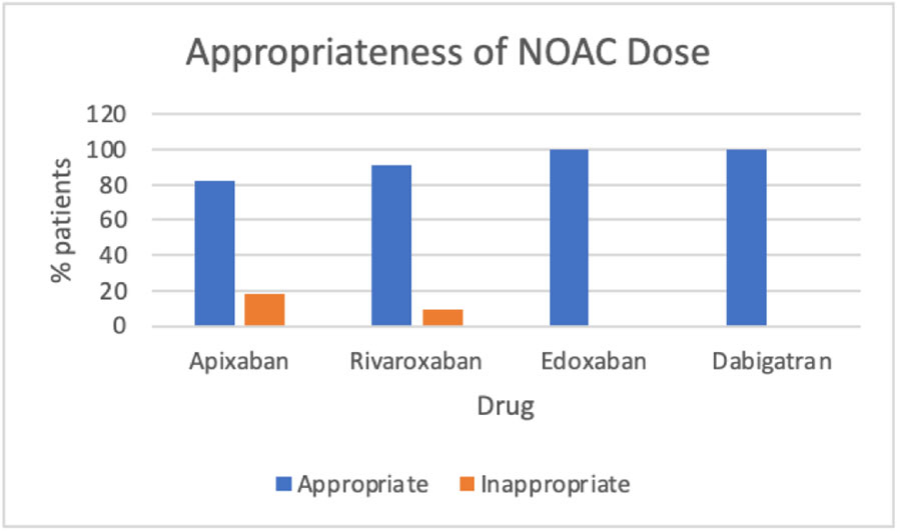
NOAC Dose Appropriateness

**Fig. 2 (abstract A27).**
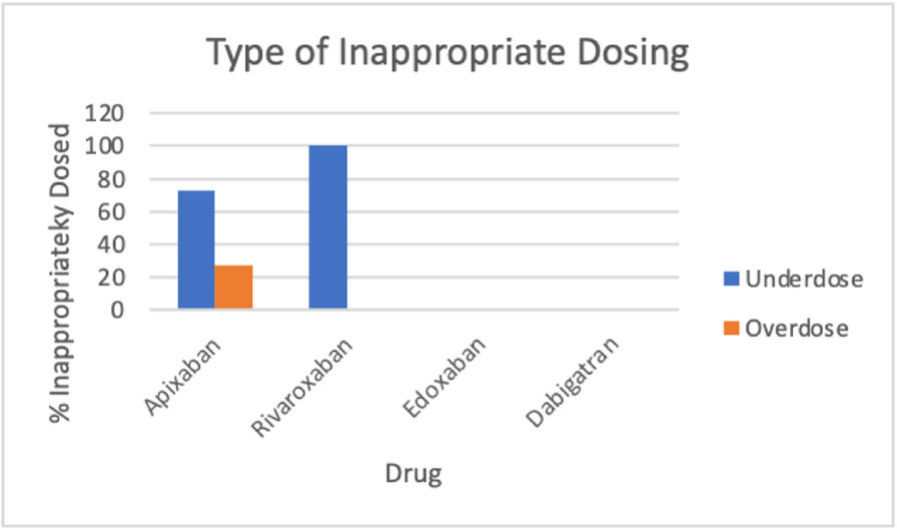
Characteristics of Inappropriate Dosing

**Fig. 3 (abstract A27).**
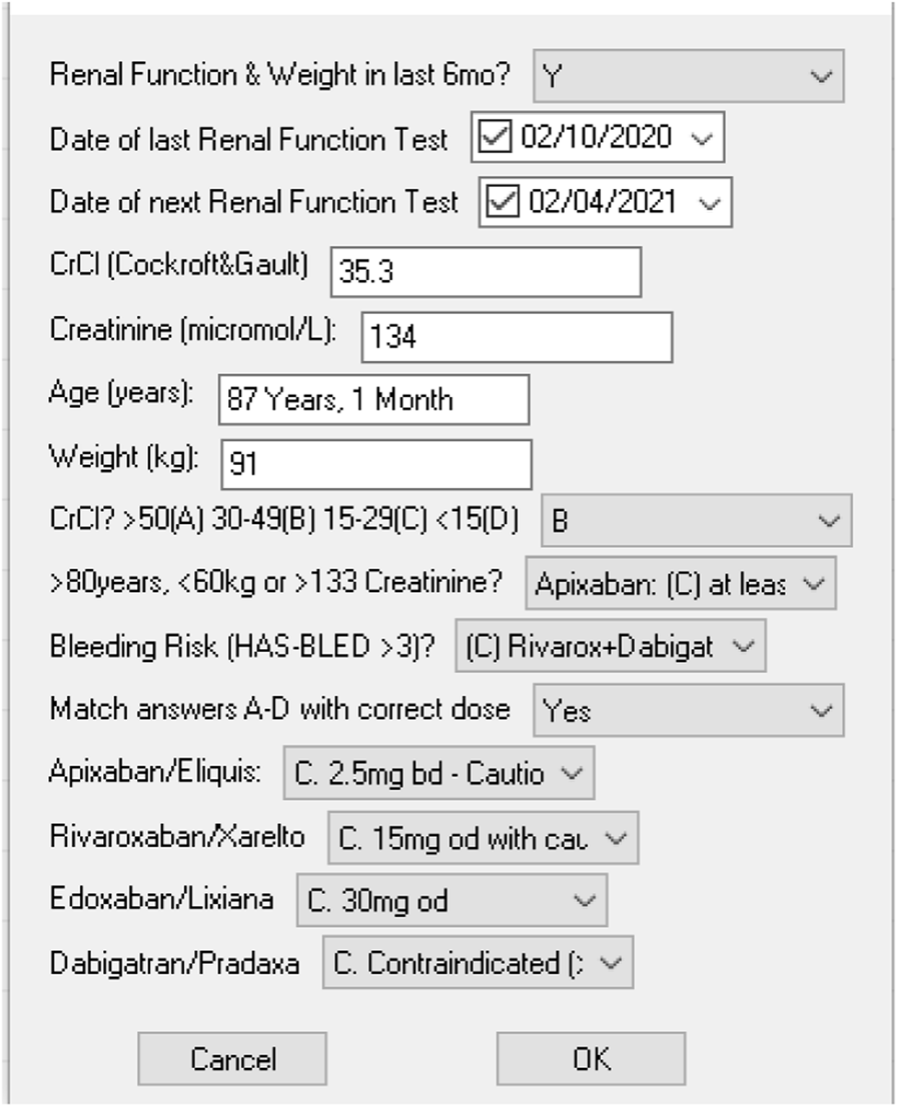
Sample Entry of CGP Computerised NOAC Dosing Aid

**Fig. 4 (abstract A27).**
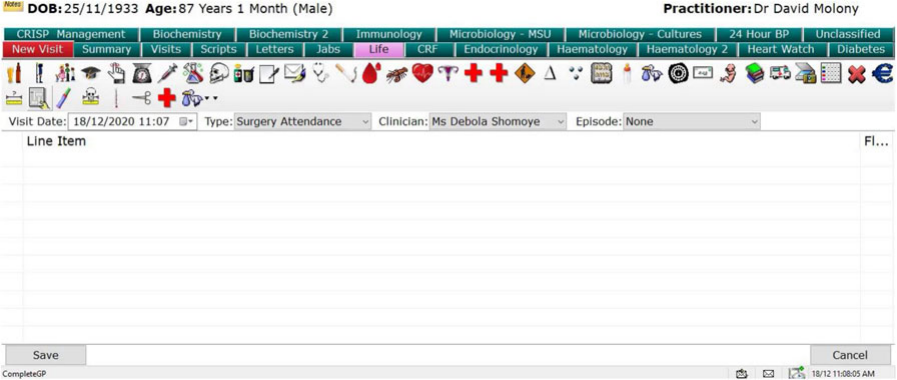
Preview of CGP Computerised NOAC Dosing Aid

### A28: Designing an e-health portal for the patient voice in cancer research to promote public and patient involvement in research across academic and patient communities

#### Aoife Gordon^1^, David Gomez^2^, Tom Hope^2^, Walter Kolch^1^, Teresa McNally^2^, Éidín Ní Shé^4^, Mary Staunton^2^, Tina O’Sullivan^2^, Antoinette Perry^5^, Romina Silva^2^, Husvinee Sudaramurthi^3^, Ramon Whelan^2^, Amanda McCann^1,2^, Elaine Quinn^2^

##### ^1^UCD School of Medicine, University College Dublin, Belfield, Dublin 4; ^2^Patient Voice in Cancer Research (PVCR), UCD Conway Institute of Biomolecular and Biomedical Research, University College Dublin, Belfield, Dublin 4; ^3^UCD School of Biomolecular and Biomedical Sciences, UCD Conway Institute, University College Dublin, Belfield, Dublin 4; ^4^UCD School of Nursing, Midwifery and Health Systems, University College Dublin, Belfield, Dublin 4; ^5^School of Biology and Environmental Science, Science West, O’Brien Science Centre, University College Dublin, Belfield, Dublin 4

**Background**

The Patient Voice in Cancer Research (PVCR) grew from an “*unmet need”* to involve the voices of cancer patients and their families with the scientific research process. To bring the PVCR in line with Public Patient Involvement (PPI) initiatives internationally, a dedicated, engaging website was drafted as a vital communication tool.

**Materials and methods**

A literature review of international PPI websites (Canada, Europe, UK) and attendance at the annual PPI Summer School (University of Limerick) were undertaken. A consent form and interview questions for PVCR Committee Members were designed and interviews conducted with cancer patients (6) and researchers (5) to develop 11 informative / reflective profiles. The National Adult Literacy Agency guidance was sought concerning requirements for obtaining a *Plain English Mark,* in line with international standards.

**Results**

The profiles and transcripts have been reviewed, and permission granted for use in the PVCR website, social media and a manuscript for peer review entitled ‘*Could You Give Us An Idea On What We Are All Doing Here?*’. This paper documents the PVCR journey from initiation to involvement nationally. and is being prepared for the journal, *Research Involvement and Engagement*.

**Conclusions**

The PVCR are paving a new path in PPI in cancer research in Ireland. This research included assimilating (i) a reflective insight into the motivation and impact of PVCR membership since its initiation in 2016 and (ii) the vision of patients and researchers for PPI and its importance in advancing cancer research; crucial aspects for the PVCR website.

**Acknowledgement**

*The authors would like to acknowledge that this project would not have been possible without the Patient Voice in Cancer Research (PVCR) committee members and cancer researchers within the UCD Conway Institute and Systems Biology Ireland (SBI).*

### A29: What is the benefit of adjuvant therapy for patients who have undergone neoadjuvant therapy for oesophageal adenocarcinoma?

#### Shantam Agarwal^1^, Yuriy Dovhan^1^, Diane Doran^1^, Ciara Owens^1^, Magdalina Fadel^1^, Diana Williams^1^, Claire Donohoe^2^

##### ^1^School of Medicine, Trinity College Dublin, Dublin 2, Ireland; ^2^Department of Surgery, Trinity College Dublin, Dublin 2, Ireland

###### **Correspondence:** Shantam Agarwal (agarwash@tcd.ie)

**Background**

Oesophageal adenocarcinoma (OAC) is an aggressive cancer with worse prognosis in the advanced stage of disease [1]. Standard treatment for locally advanced oesophageal cancer is neoadjuvant chemoradiation followed by surgery (trimodal regimen) [2]. The role of postoperative treatment is unclear [2]. Our study aims to review the benefits of adjuvant therapy in the postoperative period.

**Methods**

We carried out a review of 8 papers using the following databases: PubMed, Embase, clinical trials.gov, Cochrane. We set out to determine the hazard ratio, average 3-year and 5-year overall survival percentages of patients who underwent both neoadjuvant and adjuvant therapy.

**Results**

We identified a total of 16,299 patients. Of this cohort, 1712 underwent adjuvant therapy (experimental arm) and the remaining 14,587 were treated in the control arm. There was a 15% reduction in death (HR - 0.85) with the inclusion of adjuvant therapy. Average 5-year OS was 38.4% in the experimental arm in comparison to the control arm - 33.8%; P value - 0.42. Average 3-year OS was 50.9% in the experimental arm and 45.9% in the control arm; P value - 0.44. Additionally, a 30%, 26% and 20% reduction in risk of death was observed in node positive OAC patients in the Burt et al. (2017), Eng et al. (2018) and Nevala-Plagemann et al (2018) randomized controlled trials respectively.

**Conclusion**

Although a considerable benefit is seen with the inclusion of this therapy to the trimodal regimen, its use across a range of disease presentations did not present any statistically significant benefit in context of both 3 and 5-year overall survival rates. Nevertheless, a promising finding of reduced risk of death (statistically significant) was observed in the residual nodal disease cohort. This trial dispenses a great opportunity for considerable research to be conducted in this area to definitively conclude if it should be incorporated into the typical treatment regime and thus combat the high mortality rate associated with this disease.

**References**

1. Pennathur A, Gibson MK, Jobe BA, Luketich JD. Oesophageal carcinoma. The Lancet. 2013;381(9864):400–12.

2. Ajani JA, D’Amico TA, Bentrem DJ, Chao J, Corvera C, Das P, et al. Esophageal and Esophagogastric Junction Cancers, Version 2.2019, NCCN Clinical Practice Guidelines in Oncology. Journal of the National Comprehensive Cancer Network. 2019;17(7):855–83. Eng O, Nelson R, Konstantinidis I, Chao J, Erhunmwunsee L, Raz D et al. Disparities in survival after trimodality therapy for esophageal adenocarcinoma. Diseases of the Esophagus. 2018; 31(9).

3. Burt BM, Groth SS, Sada YH, Farjah F, Cornwell L, Sugarbaker DJ, et al. Utility of Adjuvant Chemotherapy After Neoadjuvant Chemoradiation and Esophagectomy for Esophageal Cancer. Annals of Surgery. 2017;266(2):297–304.

4. Eng OS, Nelson RA, Konstantinidis I, Chao J, Erhunmwunsee L, Raz DJ, et al. Disparities in survival after trimodality therapy for esophageal adenocarcinoma. Diseases of the Esophagus. 2018;31(9).

5. Nevala-Plagemann C, Francis S, Cavalieri C, Tao R, Whisenant J, Glasgow R, et al. Benefit of adjuvant chemotherapy based on lymph node involvement for oesophageal cancer following trimodality therapy. ESMO Open. 2018;3(5).

### A30: Road traffic collision related injuries in a national rehabilitation hospital; a 5-Year retrospective review

#### Aisling O’Keeffe^1^, Áine Carroll^1,2^

##### ^1^School of Medicine, University College Dublin, Ireland; ^2^Academic Department, National Rehabilitation Hospital Dublin, Ireland

###### **Correspondence:** Aisling O’Keeffe (aisling.okeeffe@ucdconnect.ie)

**Background**

The Road Safety Authority (RSA) came into being on 1 September 2006 as a statutory organisation created by the Road Safety Authority Act, 2006 with the aim to reduce collisions, deaths, and injuries. Since then, the number of deaths on the roads has reduced but according to The Major Trauma Audit (MTA) National Reports, many are surviving road traffic collisions with life altering injuries. We decided to undertake a review to see if the presentations to the National Rehabilitation Hospital (NRH) of road traffic trauma related injury had changed.

**Methods**

Retrospective review of healthcare records in a Tertiary Rehabilitation Hospital. All patients discharged from the in-patient rehabilitation service with an ICD 10 coded diagnosis of Transport accidents (V00 – V89.9) (Transport accident-related injury) from 2014-2018 were included.

**Results**

338 cases were identified. 179 did not meet the inclusion criteria due to record duplication and miscoding.

The total number of healthcare records analysed systematically using a standardised proforma was 159.

109 (68%) were male and 50 32%) were female. 115 (72%) were under 40 years of age. 122 (77%) had traumatic brain injuries, 32 (20%) had traumatic spinal cord injuries and 4 (2.5%) had traumatic amputation. There were no combined injuries. There was an overall reduction in RTC related injuries over the 4 years studied but the numbers varied over each year, so an overall trend was not apparent.

**Conclusion**

The number of admissions to a National Rehabilitation Hospital with Road traffic collision related injury reduced over the course of the 5 years of review but remains elevated. Linking data between MTA and NRH admission information may be an effective way to better understand the impact of RSA strategy and policy.

**Acknowledgements**

The authors are grateful to the coding staff at the National Rehabilitation Hospital for their assistance with this project.

### A31: In patients admitted to ICU with SARS-COV2 infection, is dexamethasone superior to standard care in improving mortality? A systematic review of evidence to date

#### Laith Al Azawi^1^, Conor Farrell^1^, Lauren Hayes^1^, Liam Mariga^1^, Imad Mirza^1^, Mariam Salem^1^, Carmel Kennedy^2^

##### ^1^School of Medicine, Trinity College Dublin, Dublin 2, Ireland; ^2^Department of Pharmacology and Therapeutics, St James’s Hospital, Dublin, Ireland

###### **Correspondence:** Laith Al Azawi

**Background**

Dexamethasone is a potent broad-spectrum corticosteroid that decreases the transcription of pro- inflammatory cytokines, whilst simultaneously increasing the transcription of anti-inflammatory cytokines. The cytokine storm that is central to the pathogenesis of Acute Respiratory Distress Syndrome (ARDS) and multi- organ failure is seen in severe acute respiratory syndrome coronavirus 2 (SARS-CoV-2) related deaths. The objective of this study is to appraise the current evidence for the use of dexamethasone in the treatment of patients admitted to the intensive care unit (ICU) with SARS-COV2 infection.

**Materials and methods**

We conducted searches of two databases, EMBASE and PubMed, using the terms “COVID-19”, “Dexamethasone”, and “ICU”. The search was limited to English language publications only, and human clinical trials only. A PRISMA flow chart was used to guide our search methodology.

**Results**

The database search identified 59 articles. Of these, two duplicates were discarded, and 57 citations were screened. 54 of these publications were deemed irrelevant based on the inclusion and exclusion criteria. Three were forwarded for full text review and met inclusion and exclusion criteria on full-text review. All three were deemed eligible. The selected studies consisted of two Randomised Clinical Trials (RCTs), and one Case Series Report. The results from the three papers were unanimous in their conclusion that dexamethasone was superior to standard care in the treatment of patients admitted to ICU with SARS- COV2. There was also a shorter duration of hospitalisation seen in the patient group treated with dexamethasone.

**Conclusions**

Our systematic review found that dexamethasone was superior to standard care alone in patients admitted to the ICU with SARS-COV2 infection. However, administration of dexamethasone to patients not on respiratory support resulted in a higher incidence of death, compared to standard care alone.

### A32: Investigating the effects of substrate stiffness of biodegradable polymers on astrocyte physiology

#### Seán Kerr^1^, Cian O’Connor^2^, Adrian Dervan^2^, Maeve Caldwell^3^

##### ^1^School of Medicine, University College Dublin, Belfield, Dublin 4, Ireland; ^2^Tissue Engineering Research Group, Department of Anatomy, Royal College of Surgeons in Ireland, 123 St. Stephen's Green, Dublin 2, Ireland; ^3^Trinity College Institute of Neuroscience, Department of Physiology, Trinity College Dublin, Dublin, Ireland

###### **Correspondence:** Seán Kerr

**Background**

Astrocytes are non-neuronal supportive cells which play a number of diverse roles in the healthy central nervous system (CNS). Following spinal cord injury, astrocytes become reactive (characterized by altered gene expression and morphology [1]) and form a glial scar around the lesion [2]. This glial scar contributes to tissue softening and altered extracellular matrix (ECM) deposition in the CNS. Interestingly, both stiffness and ECM composition have been shown to affect astrocyte reactivity [3]. However, the effect of substrate stiffness on astrocytes is not yet well understood. The increasing use of biotherapeutic implantable devices has led to heightened interest in the reaction of cells to implant properties such as stiffness. Therefore, characterization of the reaction of astrocytes to substrates of varying stiffness is a critical factor to consider in the design of implantable therapeutic devices for use in the CNS.

**Methods**

We aimed to create a 3D hyaluronic acid (HyA) scaffold with tuneable stiffness. However, HyA requires trophic ECM proteins to support cell attachment. We first cultured astrocytes on coverslips with various ECM components to assess their effects on astrocyte physiology via immunocytochemistry, fluorescence microscopy and ImageJ software. We then manufactured HyA scaffolds using 3 concentrations; 3, 5 & 10mg/mL. Stiffness was assessed by mechanical testing, while pore size was characterized by Toluidine blue staining and microscopy. We then cultured astrocytes on scaffolds of varying stiffness and assessed their metabolic activity (via Alamar blue assays) and expression of reactivity associated proteins (via ELISA) across multiple timepoints.

**Results**

Astrocyte metabolic activity was not significantly altered by the assessed ECM components. However, actin outgrowth and cell area were significantly increased in astrocytes cultured on ECM1 and ECM1+2, while nuclear:cytoplasm area and GFAP intensity (basic metrics of astrocyte reactivity) were not significantly altered in any group. The manufacture of scaffolds with tuneable stiffness was successful; the 3mg/mL scaffolds were significantly softer than the 10mg/mL scaffolds. Pore size was significantly higher in the 3 and 5mg/mL scaffolds compared to the 10mg/mL scaffolds. The metabolic activity of astrocytes cultured on the scaffolds initially decreased (D1-D4), but remained consistent at later timepoints (D4-D21); the 3mg/mL scaffold displayed the greatest activity at D21. Secretion of IL-6 from the astrocytes significantly decreased in all groups from D1 onwards, with no detectable levels at D21. Quantification of actin and GFAP coverage revealed that softer scaffolds had significantly greater coverage of both actin and GFAP.

**Conclusions**

The substrate stiffness of HyA scaffolds has significant effects on astrocyte physiology and has significant implications for scaffold design.

**References**

1. Sofroniew MV. Molecular dissection of reactive astrogliosis and glial scar formation. Trends Neurosci. 2009; 32(12):638-47.

2. Cregg JM, DePaul MA, Filous AR, Lang BT, Tran A, Silver J. Functional regeneration beyond the glial scar. Exp Neurol. 2014; 253:197-207.

3. Moeendarbary E, Weber IP, Sheridan GK, Koser DE, Soleman S, Haenzi B, et al. The soft mechanical signature of glial scars in the central nervous system. Nat Commun. 2017; 8:14787.

### A33: Prolonged response to metastatic pancreatic cancer treated with pembrolizumab based on mismatch repair status characterized from next generation sequencing

#### Lan D. Ngo^1,2^, Ana C. Garrido-Castro^2^, Melissa E. Hughes^2^, Nancy U. Lin^2^

##### ^1^UCD School of Medicine, University College Dublin, Belfield, Dublin 4, Ireland; ^2^Dana-Farber Cancer Institute, 450 Brookline Ave, Boston, MA, 02215, USA

**Background**

Metastatic pancreatic cancer is associated with poor prognosis. With standard regimens, including gemcitabine/nab-paclitaxel or FOLFIRINOX, 1-year survival is 10% [1]. Patients with metastatic tumors that progress on standard treatment and have microsatellite instability-high (MSI-H) or mismatch repair-deficient (MMR-D) tumors are potential candidates for pembrolizumab, a PD-1 immune checkpoint inhibitor. The frequency of MSI-H/MMR-D tumors is 2% in pancreatic adenocarcinomas, compared to 17% in endometrial and 6% in colorectal cancers [2]. MSI/MMR status has traditionally been determined using immunohistochemistry (IHC) or PCR testing. Patients at Dana-Farber Cancer Institute are approached for OncoPanel testing, a targeted next-generation sequencing (NGS) platform, on primary or metastatic tumor tissue. Using a validated bioinformatics algorithm, MSI/MMR status can be inferred from OncoPanel data [3]. Here, we present a case report of a patient with metastatic pancreatic cancer identified as MSI-H/MMR-D using OncoPanel.

**Case report**

A 71-year-old female presented with abdominal and back pain, and a palpable left supraclavicular mass. PET imaging revealed a pancreatic mass and enlarged retroperitoneal and supraclavicular nodes. A supraclavicular node biopsy confirmed metastatic pancreatic adenocarcinoma. Genetic testing confirmed Lynch syndrome. The patient was treated with FOLFOX (progression after 5 months) and gemcitabine/nab-paclitaxel (progression after 2 months). OncoPanel testing showed MSI-H/MMR-D and confirmatory IHC on the pancreatic mass revealed absent MSH2 staining, consistent with MMR-D. The patient initiated pembrolizumab and has continued treatment for 16 months, with the most recent scan showing response.

**Conclusion**

This case illustrates prolonged clinical benefit from PD-1 inhibition in a patient with metastatic MSI-H/MMR-D pancreatic cancer refractory to standard chemotherapy. In cancer types with low frequency of MSI-H/MMR-D, institution-wide NGS platforms can be leveraged as a cost-effective method to identify MMR-D/MSI-H and potential candidates for immunotherapy.

**Consent to publish**

Patients at Dana-Farber Cancer Institute are approached for consent to a cancer research study for OncoPanel tumor testing. The participant has provided written informed consent and consent for publication.

**References**

1. Azar I, Virk G, Esfandiarifard S, et al. Treatment and survival rates of stage IV pancreatic cancer at VA hospitals: a nationwide study. J Gastrointest Oncol. 2019; 10(4): 703-711.

2. Le DT, Durham NJ, Smith NK, Wang H, Bartlett BR, Aulakh LK, et al. Mismatch-repair deficiency predicts response of solid tumors to PD-1 blockade. Science. 2017; 357(6349): 409–413.

3. Nowak JA, Yurgelun MB, Bruce JL, et al. Detection of Mismatch Repair Deficiency and Microsatellite Instability in Colorectal Adenocarcinoma by Targeted Next-Generation Sequencing. J Mol Diagn 19:84-91, 2017.

### A34: Vaccines in the age of skepticism and a pandemic

#### Jonathan L. Jeger^1‡^, Thomas Drago^1‡^, Mark Wadid^1‡^, Sean Garvey^1‡^, Kirolos Bassily^2^, John Hanna^3^

##### ^1^School of Medicine, Trinity College Dublin, Dublin, Ireland; ^2^Midlands Regional Hospital, Portlaoise, Ireland; ^3^Sligo University Hospital, Sligo, Ireland

###### **Correspondence:** Jonathan L. Jeger (jegerj@tcd.ie)

^‡^Joint first-authorship

**Background**

Vaccination was first discovered in 1796 by Edward Jenner in an effort to prevent the spread of smallpox disease [1,2]. The smallpox vaccine, described as the most successful vaccination effort in human history, was administered for over 150 years until the World Health Organization (WHO) declared the disease eradicated in 1980 [3,4,5]. In 1998, a study by Wakefield et al. reported a possible causal relationship between measles-mumps-rubella vaccination and autism in 12 children in the UK, resulting in the anti-immunization lobby receiving considerable media attention [6,7]. Although the publication was retracted in 2010, the controversy surrounding vaccination has had a lasting impact [8]. With COVID-19 cases still increasing throughout Ireland and the rest of the world, and COVID-19 vaccination options on the horizon, we find ourselves at an important moment in time to take a closer look at vaccination and the anti-immunization lobby.

**Materials and methods**

We performed a literature search with regards to the COVID-19 vaccination options, and have explored current vaccine trials and data. We then compared our findings with the framework for vaccine allocation and prioritization as outlined by the WHO. Additionally, we also explored the ethical question of who should receive access to vaccines first.

**Results**

After extensive review of the literature, we gathered information regarding the importance of vaccinations, herd immunity, and the strategic ways to immunize large populations. By highlighting the statistically significant results of several vaccine candidates, we illustrated the importance of the vaccination process as a safe and effective way to combat a virus in the midst of a pandemic affecting nearly every nation worldwide. Through careful research and analysis, our study aims to not only reduce skepticism of these vaccines, but to also emphasize the statistics that highlight their benefits.

**Conclusion**

Skepticism of a vaccine for a virus reaching all ends of the globe is a profound issue seen in countless nations. With growing concerns about safety and efficacy, it is important to delineate data emphasizing the benefits of multiple tested therapeutics in order to effectively combat the pandemic.

**References**

1. Begg N, Nicoll A. Myths in medicine: immunisation. BMJ. 1994; 309(6961):1073-1075.

2. Stewart AJ, Devlin PM. The history of the smallpox vaccine. Journal of Infection. 2006; 52(5):329-334.

3. Maurer DM, Harrington BC, Lane MJ. Smallpox vaccine: contraindications, administration, and adverse reactions. American family physician. 2003; 68(5):889-896.

4. Henderson DA, Inglesby TV, Bartlett JG, Ascher MS, Eitzen E, Jahrling PB, Hauer J, Layton M, McDade J, Osterholm MT, O'Toole T. Smallpox as a biological weapon: medical and public health management. JAMA. 1999; 281(22):2127-2137.

5. World Health Organization. The global eradication of smallpox: final report of the Global Commission for the Certification of Smallpox Eradication [Internet]. Geneva: Switzerland; 1979 [cited 2020 Dec 1]. Available from: https://apps.who.int/iris/handle/10665/39253

6. Wakefield AJ, Murch SH, Anthony A, Linnell J, Casson DM, Malik M, Berelowitz M, Dhillon AP, Thomson MA, Harvey P, Valentine A. RETRACTED: Ileal-lymphoid-nodular hyperplasia, non-specific colitis, and pervasive developmental disorder in children. The Lancet. 1998; 351(9103):637-641.

7. Burgess DC, Burgess MA, Leask J. The MMR vaccination and autism controversy in United Kingdom 1998–2005: Inevitable community outrage or a failure of risk communication?. Vaccine. 2006; 24(18):3921-3928.

8. Caplan AL. Retraction—Ileal-lymphoid-nodular hyperplasia, non-specific colitis, and pervasive developmental disorder in children. The Weekly Epidemiological Record. 2010; 84:301-308.

### A35: Post-study point-of-care oral fluid testing in HIV-1 vaccines

#### Karina Oganezova^1,2^, Elvin J. Fontana-Martinez^2^, Jon A. Gothing^2^, Alisha Pandit^2^, Esther Kwara^3^, Katherine Yanosick^4^, Joan Dragavon^5^, Erin A. Goecker^5^, Janine Maenza^6,7^, Nicole Espy^6^, Frank Tomaka^8^, Ludo Lavreys^9^, Mary Allen^10^, Patricia D’Souza^10^, John Hural^6^, Robert W. Coombs^5,7^, Raphael Dolin^4,11^, Michael S. Seaman^4,11^, Stephen R. Walsh^2,11^, Lindsey R. Baden^2,11^

##### ^1^School of Medicine, Trinity College, Dublin, Ireland; ^2^Division of Infectious Diseases, Brigham and Women’s Hospital, Boston, MA, USA; ^3^Morehouse School of Medicine, Atlanta, GA, USA; ^4^Center for Virology and Vaccine Research, Beth Israel Deaconess Medical Center, Boston, MA, USA; ^5^Department of Laboratory Medicine, University of Washington, Seattle, WA, USA; ^6^Vaccine and Infectious Disease Division, Fred Hutchinson Cancer Research Center, Seattle, WA, USA; ^7^Department of Medicine, University of Washington, Seattle, WA, USA; ^8^Janssen Pharmaceutical Research and Development, Titusville, NJ, USA; ^9^Janssen Vaccines & Prevention, B.V., Leiden, The Netherlands; ^10^National Institute of Allergy and Infectious Diseases, Rockville, MD, USA; ^11^Harvard Medical School, Boston, MA, USA

###### **Correspondence:** Karina Oganezova

**Background**

Experimental HIV-1 vaccines frequently elicit antibodies against HIV-1 which may react with commonly used HIV diagnostic tests, a phenomenon known as vaccine-induced seropositivity/seroreactivity (VISP/VISR). We sought to determine under clinic conditions if the OraQuick ADVANCE Rapid HIV-1/2 Antibody Test could detect HIV-1 vaccine-induced antibodies.

**Materials and methods**

Plasma assessment of HIV-1 cross-reactivity was examined in end-of-study samples from 57 healthy, HIV-uninfected participants who received a candidate vaccine which has entered Phase 2B and 3 testing. We also screened 120 healthy, HIV-uninfected, unblinded HIV-1 vaccine participants with VISP/VISR for an assessment using saliva. These participants came from 21 different parent vaccine protocols representing 17 different vaccine regimens, all of which contained an HIV-1 envelope immunogen. OraQuick ADVANCE was compared to results from concurrent blood samples using a series of commercial HIV screening immunoassays.

**Results**

Fifty-seven unique plasma samples from vaccine recipients were assayed *in vitro* and only one (1.8%) was reactive by OraQuick ADVANCE (Table 1). None of the 120 clinic participants (0%, [95% CI 0% to 3.7%]) tested positive by OraQuick ADVANCE and all were confirmed to be uninfected by HIV-1 viral RNA testing. 118 of the 120 (98.3%) participants had a reactive HIV test for VISP/VISR: 77 (64%) had at least one reactive fourth-generation HIV-1 diagnostic test (p<0.0001 vs no reactive OraQuick ADVANCE results) and 41 (34%) only had a reactive test by the less specific third-generation Abbott Prism assay (p<0.0001 vs no reactive OraQuick ADVANCE results) (Table 2).

**Conclusions**

These data suggest that this test has limited reactivity to HIV-1 antibodies elicited by these candidate HIV-1 vaccines. The related over-the-counter patient-controlled OraQuick In-Home test, while not tested in this study, may potentially provide similar results. Due to the limited sensitivity of both OraQuick ADVANCE and OraQuick In-Home HIV Test for detection of acute HIV, we suggest that these tests continue to be paired with comprehensive pre-test and post-test HIV counselling.

**Table 1 (abstract A35).** Analysis of OraQuick ADVANCE cross-reactivity with plasma samples from vaccine recipients

HIV-1 Diagnostic Test	Participants Testing Reactive n (%)	p value vs OraQuick ADVANCE
OraQuick ADVANCE	1 of 57 (1.8%)	----
BioRad GS	53 of 57 (93%)	< 0.0001
Abbott Architect	54 of 57 (95%)	< 0.0001
Alere Determine	52 of 57 (91%)	< 0.0001
Abbott Prism^1^	1 of 1 (100%)	----

^1^ Abbott Prism was only used if samples tested negative on all three of the fourth-generation antigen/antibody tests (n=15)

**Table 2 (abstract A35).** Analysis of OraQuick ADVANCE saliva cross-reactivity with blood tests

HIV-1 Diagnostic Test	Participants Testing Reactive n (%)	p value vs OraQuick ADVANCE
OraQuick ADVANCE	0 of 120 (0%)	----
BioRad GS	65 of 120 (54%)	< 0.0001
Abbott Architect	76 of 120 (63%)	< 0.0001
Alere Determine	58 of 120 (48%)	< 0.0001
Abbott Prism^1^	41 of 43 (95%)	< 0.0001

^1^ Abbott Prism was only used if samples tested negative on all three of the fourth-generation antigen/antibody tests (n=41)

### A36: The impact of the SARS-CoV-2 pandemic on referral characteristics to a national tertiary spinal injuries unit

#### Louis O’Halloran^1^, Daniel P Ahern^2,3^, Jake M McDonnell^4^, Michael K Dodds^3^, Frank Lyons^3^, Noelle Cassidy,^3^ Marcus Timlin^3^, Seamus Morris^3^, Keith Synnott^3^, Joseph S Butler^3,5^

##### ^1^School of Medicine, University College Dublin, Dublin, Ireland; ^2^School of Medicine, Trinity College Dublin, Dublin, Ireland; ^3^National Spinal Injuries Unit, Department of Trauma & Orthopaedic Surgery, Mater Misericordiae University Hospital, Dublin, Ireland; ^4^Royal College of Surgeons in Ireland, Dublin, Ireland; ^5^UCD Clinical Research Centre, UCD School of Medicine, Mater Misericordiae University Hospital, Dublin, Ireland

Conflict of Interest: The authors declare no conflict of interest.

**Background:**

The SARS-CoV-2 pandemic has had profound implications on healthcare institutions. The aim of this study is to assess and compare referral patterns during Covid-19 to corresponding dates for the preceding three years (2017-2019), in order to pre-emptively coordinate the logistics of the surgical unit for similar future experiences.

**Methods:**

A retrospective review was carried out at our institution, a national tertiary referral centre for spine pathology. Two distinct time-points were chosen to represent the varied levels of social restriction during the current pandemic; (i) Study period 1 (SP1) from 11/03/20-08/06/20 represents a national lockdown, and (ii) Study period 2 (SP2) from 09/06/20-09/09/20 indicates an easing of restrictions. Both periods were compared to corresponding dates (CP1: 11/03-08/06 and CP2: 09/06-09/09) for the preceding three years (2017-2019). Data collected included age, gender, and mechanism of injury (MOI) for descriptive analyses. MOIs were categorised into disc disease, cyclist, road-traffic-accident (RTA), falls < 2m, falls > 2m, malignancy, sporting injuries, and miscellaneous.

**Results:**

All MOI categories witnessed a reduction in referral numbers during SP1; disc disease (-29%), cyclist (-5%), RTAs (-66%), Falls <2m (-39%), Falls > 2m (-17%), malignancy (-33%), sporting injuries (-100%), miscellaneous (-58%). 4/8 categories (RTAs, falls < 2m, malignancy, miscellaneous) showed a trend towards return of pre-lockdown values during SP2. Two categories (disc disease, falls >2m) showed a further reduction (-34%, -27%) during SP2. One category (sporting injuries) portrayed a complete return to normal values during SP2 while a notable increase in cyclist related referrals were witnessed (+63%) when compared with corresponding dates of previous years.

**Conclusion:**

Spinal injury continues to occur across almost all categories, albeit at considerably reduced numbers. RTAs and falls remained the most common mechanism of injury. Awareness needs to be drawn to the reduction of malignancy related referrals to dissuade people with such symptoms from avoiding presentation to hospital over periods of social restrictions.

### A37: Idiopathic intracranial hypertension and anemia: A systematic review, meta-analysis and case series

#### Ethan Waisberg^1^, Caberry W. Yu^2^, Jonathan A. Micieli^3^

##### ^1^UCD School of Medicine, University College Dublin, Belfield, Dublin 4, Ireland; ^2^School of Medicine, Faculty of Health Sciences, Queen’s University, Kingston, Ontario, Canada; ^3^Department of Ophthalmology and Vision Sciences, University of Toronto, Toronto, Ontario, Canada

###### **Correspondence:** Ethan Waisberg

**Background**

Idiopathic intracranial hypertension (IIH) is defined as elevated intracranial pressure in the absence of an identifiable cause. The relationship between IIH and anemia remains controversial. The goal of this study was to report on consecutive cases of IIH with a fulminant course and severe anemia (hemoglobin <80g/L) to provide additional evidence to this topic. This study also aimed to examine the causal relationship between anemia and IIH.

**Materials and Methods**

The retrospective case series of four patients with IIH and severe anemia, was diagnosed after neurologic imaging, lumbar puncture and an extensive workup to exclude secondary causes. MEDLINE, Embase, Cochrane Library, and grey literature were searched to September 2020. Primary studies on patients with diagnoses of anemia of any kind and IIH were included. Primary outcomes included the total number of cases of anemia and IIH. A meta-analysis on the prevalence of anemia in IIH compared to control patients was conducted.

**Results**

Four patients (all female) were included in the series with an average age of 28 years, and mean body mass index of 32.2kg/m^2^. Visual acuity ranged from 20/20 to 20/50 and the average Humphrey mean deviation was -7.30dB. The average hemoglobin at presentation was 71g/L and MCV 72.0. Correction of the anemia required aggressive treatment with intravenous iron in two patients and oral iron in the remaining cases. All patients were also treated with acetazolamide and optic nerve sheath fenestration for one patient. All patients had resolution of the papilledema and symptoms within 3 months as their hemoglobin increased to normal levels at that time.

Overall, 74 cases and 5 observational or case-control studies were included. Pooled incidence of anemia in IIH patients was 195/1073 (18.2%). IIH patients (n=774) had a significantly higher prevalence of anemia compared to controls (n=230,981) (RR 1.44 [95% CI 1.08, 1.92]). Of the reported patients, 35/59 (59.3%) showed improvement or resolution with anemia treatment only, 7/59 (11.9%) with intracranial pressure-lowering therapy only, and 15/59 (25.4%) with both.

**Conclusions**

Anemia is 44% more common in IIH compared to control patients, with many case reports suggesting a direct relationship. A complete blood count is important in the workup of patients presenting with papilledema, especially if there are severe peripapillary cotton wool spots. Our case series suggests a direct relationship between severe anemia and fulminant IIH as our patients had severe anemia at onset and resolution of symptoms and papilledema after normalization of hemoglobin.

### A38: Medical screening in mental health emergencies

#### Fouad Helmy^1^, Nigel Salter^2^

##### ^1^UCD School of Medicine, Belfield, Dublin 4, Ireland; ^2^Emergency Department, St. Vincents University hospital, Elm Park, Dublin 4, Ireland

###### **Correspondence:** Fouad Helmy

**Background**

Mental health presentations account for about 5% of Emergency Department (ED) attendance but requires higher level of resources. Often such patients present in an acute crisis triggered by a life changing event necessitating a multidisciplinary input which causes their management plan to be complex and slow. Determining a medical illness as the cause of a patient’s acute psychiatric symptoms is not always straightforward and limited de-escalation facilities in the ED can lead to further inefficiencies in patient care. Despite this, there is currently limited literature on the flow of patients in the ED with psychiatric presentations. This pilot project aims to analyse the current practice of medical assessments in the ED of such patients and their effects on patient flow.

**Methods**

Patients were registered, triaged, and clinical notes documented on SVUH ED IT system: IMS MAXIMS. Data was gathered for 156 ED attendances over a 10-week consecutive period on investigations performed (including bloods, ECG, CT-Brain, Urine Toxicology, Urine Dipstick, and X-ray), total ED visit duration (Figure 1), and significant testing performed.

**Results**

119 patients (76.3% of total) had some form of investigations performed including bloods, ECG, X-rays, CT-brain, urine toxicology, and urine dipstick. The percentage of abnormal test results from total amount tested for urine dipstick and urine toxicology tests was 46.5% and 66.7% respectively. In contrast, X-rays, CT-brain, and ECGs there was a low percentage of abnormal test results - 12.5%, 7.69%, and 2.6% respectively. We compared the mean ED duration to each of the tests done and noticed, when using the Kruskal-Wallis test, that there is a significant difference in the mean ED duration when urine toxicology (p=0.005), CT-brain (p=0.002), and ECGs (p=0.011) were performed but no significance for urine dipstick testing (p=0.122). However, when we compared the mean ED duration with “abnormal” results and “not done” urine dipstick tests using Mann-Whittney U test we obtained a p value of 0.04 which is significant.

**Conclusions**

We observed a high rate of investigations being performed for patients presenting with psychiatric complaints, with a high rate of normal results for most tests. We also observed a significantly longer duration of ED stay for patients when X-rays, ECGs, and CT-brain were performed. Strategies focused on rationalising the use of investigations in the ED will optimise flow for this patient cohort.

**Fig. 1 (abstract A38).**
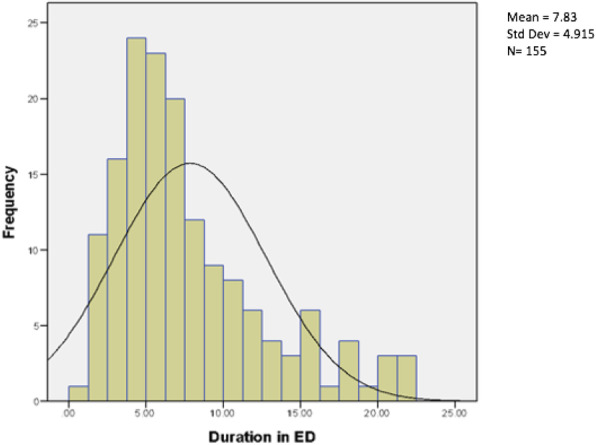
Frequency of all patients’ ED duration in hours. Line shows the normal

### A39: A retrospective study of body mass index and weight loss in Irish adults admitted to an acute hospital following infection with SARS-CoV-2

#### Fiona Newsome^1^, Ciara Murphy^2^

##### ^1^University College Dublin, Ireland; ^2^St Michaels Hospital, Dun Laoghaire, Dublin, Ireland

###### **Correspondence:** Fiona Newsome

**Background**

The rapid spread of the virus SARS-CoV-2, hereafter COVID-19, resulted in a global pandemic in March 2020. As with most infections, various complications and lengthy hospital stays have been noted in patients contracting COVID-19. Weight loss and malnutrition may be predictors for complications during the recovery from infection and have also been predictive of an increased length of hospital stay [1]. The aim of the current study was to investigate the potential associations between body mass index (BMI) and percentage weight loss with length of stay (LOS) and complications experienced as an inpatient with COVID-19.

**Materials and Methods**

This was a single centre cohort study involving adult patients diagnosed with COVID-19 and admitted to an acute general hospital in Ireland between the 1^st^ of March 2020 and the 31^st^ of May 2020. Data were extracted from relevant patient charts using a standardised reporting form and were analysed using SPSS (IBM SPSS version 26.0)

**Results**

Of the 30 patients included in the study, 29 were discharged following recovery from COVID-19 and there was one fatality. During the hospital stay, 37% (n=11) of patients suffered severe weight loss, 10% (n=3) suffered moderate weight loss, 13% (n=4) sustained mild weight loss and 40% (n=12) had no weight loss. The mean percentage weight lost was 2.91% (median, interquartile range =1.43%, 0-16.6) There was no significant relationship between percentage weight loss and LOS overall. However, in patients with a non-complex discharge (n= 20) a significant positive linear correlation (r= 0.516, p= 0.02) was found. A significant positive correlation between percentage weight loss and number of complications during recovery was also observed overall (r= 0.423, p= 0.02).

**Conclusion**

There were significant findings for both the number of complications experienced and LOS based on the percentage weight loss suffered by patients. The current study found that the larger the deficit between a patient’s weight at admission and at discharge, the greater the potential for complications and lengthier hospital stays. Therefore, the results of the current study highlight the need for appropriate multi-disciplinary management of patients with COVID-19, including targeted nutritional management during a hospital stay and following discharge. In conclusion, preventing weight loss and malnutrition is imperative in patients who are hospitalised with SARS-CoV-2 to minimise the risk of complications.

**Reference**

1. Casaer M, Van den Berghe G. Nutrition in the Acute Phase of Critical Illness. N Engl J Med. 2014;370(13):1227-1236.

### A40: The clinical significance and burden of thyroid nodules discovered incidentally

#### Rohil Dureja^1^, Caoimhe Casey^2^, Josephine Barry^3^, and Antoinette Tuthill^2^

##### ^1^School of Medicine, University College Cork, Cork, Ireland; ^2^Deperatment of Endocrinology, Cork University Hospital, Cork, Ireland; ^3^Deperatment of Radiology, Cork University Hospital, Cork, Ireland

###### **Correspondence:** Rohil Dureja

**Background**

Reporting of thyroid incidentalomas (TI) on imaging preformed for other indications has led to a clinical dilemma. The majority of thyroid nodules are benign, however; current guidelines suggest that a TI should be worked up to rule out malignancy. This study aims to determine the incidence of TIs and the likelihood that they reveal a sinister pathology in the largest Irish cohort studied to date.

**Materials & Methods**

A retrospective observational chart review was conducted using imaging studies obtained in Cork University Hospital from July 2018 to December 2018. The PACS database was searched using the defined inclusion and exclusion criteria. The body and summary of 500 carotid dopplers and 500 computed tomography (CT) thorax were manually screened for phrases such as “thyroid mass” or “thyroid nodule”. Once all the patients with TIs were identified, electronic records and their medical charts were used to track the follow ups and final outcome of the identified nodules.

**Results**

Out of 1,000 scans, 14 (1.4%) thyroid incidentalomas were discovered. The average age for TIs according to this study was 68 and an equal distribution was seen amongst the genders. The occurrence of TIs by imaging was 2/500 (0.4%) for carotid doppler and 12/500 (2.4%) for CT thorax. Three (21.4%) TIs were further evaluated with a subsequent ultrasound. All three TIs were found to be ≥ 1.0cm and underwent investigation by fine needle aspiration. Using cytology, these TIs were given a Thy 2 grading (non-neoplastic).

**Conclusion**

This study found no clinical benefit to reporting the presence of TIs discovered incidentally. The three TIs that were evaluated were found to be benign, suggesting that TIs are unlikely to have a sinister pathology. A higher number of TIs were discovered on CT in comparison to US. This can be explained by a lack of formal guidelines for reporting thyroids on CTs. The concern remains that ~98 TIs are expected to be found on these modalities in a year, this number may be enough to cause strain on the healthcare system. In addition, further work-up of these TIs burden patients with invasive investigations and cause them unnecessary anxiety about their health.

### A41: Repurposing psychological interventions for healthcare workers during COVID-19

#### Sean Treacy^1^, Tamara Schloemer^2^, Fiona McNicholas^3^, John Hayden^4^

##### ^1^School of Medicine and Medical Science, University College Dublin, Dublin, Ireland; ^2^Department of International Health, Faculty of Health, Medicine and Life Sciences, CAPHRI–Care and Public Health Research Institute, Maastricht University, Postbus 616, 6200 MD Maastricht, The Netherlands; ^3^Department of child & adolescent psychiatry, School of Medicine and Medical Science, University College Dublin, Dublin; ^4^Royal College of Surgeons in Ireland, School of Pharmacy, 111 St Stephen's Green, Dublin, Ireland

**Background**

The COVID-19 Pandemic has been shown to have a large negative impact on the mental health of healthcare workers. Evidence-based interventions that could be used to mitigate this are lacking in the literature. This systematic review aims to evaluate psychological interventions used for employees following disasters and assess the transferability of these interventions to a healthcare setting during the COVID-19 pandemic.

**Materials & Methods**

Electronic Database Embase was searched from 2015 to 2020. Studies identified alongside studies received from a previous review [1] were assessed for transferability using a checklist based on the PIET-T process model [2].

**Results**

An additional three studies were identified in the updated literature search. Eighteen studies were included for assessment of transferability. Interventions evaluated included psychological debriefing, meditation courses, cognitive behavioural therapy, mental health training courses/psychoeducation courses and Trauma Risk Management (TRiM).

**Conclusions**

TRiM could improve help seeking behaviour in healthcare workers. Meditation courses could alleviate stress in healthcare workers. Mental health training courses could build resilience in healthcare workers. Psychological debriefing has potential negative effects and is not recommended for transfer. More research needs to be undertaken in this area to assess the transferability of these interventions.

**References**

1. Brooks SK, Dunn R, Amlôt R, Greenberg N, Rubin GJ. Training and post-disaster interventions for the psychological impacts on disaster-exposed employees: a systematic review. J Ment Health. 2018:1-25.

2. Schloemer T, Schroder-Back P. Criteria for evaluating transferability of health interventions: a systematic review and thematic synthesis. Implement Sci. 2018;13(1):88.

### A42: The efficacy of SSRIs in the elderly with depression

#### Salman, Omar

##### University of Limerick School of Medicine, Limerick, Ireland

**Background**

Depression in the elderly can be challenging to treat but is known to respond well to pharmacotherapy. Selective Serotonin Reuptake Inhibitors or SSRIs are the most prescribed antidepressants because of their efficacy and low side effect profile. Little is known about direct comparisons between different SSRIs and their efficacy level. The primary outcome of this literature review is to assess the data on escitalopram, citalopram, fluoxetine, and sertraline to determine which one is the most efficacious regarding treatment of late-life depression

**Materials and methods**

The literature search was conducted using MEDLINE with full text, APAPsycArticles, and APAPsycINFO along with keywords “escitalopram”, “citalopram”, “sertraline”, “fluoxetine”, “depression”, “elderly””, and “geriatric”. This review includes studies that looked at the efficacy of SSRIs in treatment of depression in the elderly where the term “elderly” was defined as anyone aged over 60. Exclusion criteria includes studies that did not mention efficacy or look at patients under the age of 60. Studies with missing data were also excluded. Efficacy was identified using standardized depression scales such as the Hamilton Depression Scale (HAMD) [1], Geriatric Depression Scale (GDS) [2], or Montgomery-Asberg-Depression Scale (MADRS) [3]. Relevant literature was screened based on abstracts and titles. The articles were then read thoroughly, and their references were also searched to find eligibility. The results of the literature search are summarised in Figure 1.

**Results**

The use of citalopram in elderly patients with depression was assessed in 496 patients. The results are summarised in Table 1. Citalopram was associated with a greater proportion of remission with greater daily dose and HAMD scores from baseline significantly improved [4].

Escitalopram was associated with a less positive response in elderly patients with major depression. Sertraline favored poorly when compared with other antidepressants like fluvoxamine. Fluoxetine had improved efficacy compared with placebo but similar efficacy when compared with amitriptyline

**Conclusion**

Citalopram consistently showed improvement in scores of efficacy in the studies that were reviewed. There is, however, more data required in direct comparisons between SSRIs and treatment of depression in the elderly before a more concrete conclusion can be drawn.

**References**

1. Hamilton, M. A RATING SCALE FOR DEPRESSION. Journal of Neurology, Neurosurgery, and Psychiatry, 1960, 23(1), pp.56-62.

2. Yesavage, J.A. et al., Development and validation of a geriatric depression screening scale: A preliminary report. Journal of Psychiatric Research, 1982, 17(1), pp.37-49.

3. Montgomery, S.A. and Asberg, M. A New Depression Scale Designed to be Sensitive to Change, 1979, 134(4), pp.382-389.

4. Lavretsky, H. et al., Citalopram, Methylphenidate, or Their Combination in Geriatric Depression: A Randomized, Double-Blind, Placebo-Controlled Trial, 2015, American Journal of Psychiatry, 172(6), pp.561-569.

5. Kyle, C.J. et al., Comparison of the tolerability and efficacy of citalopram and amitriptyline in elderly depressed patients treated in general practice. Depression and Anxiety, 8(4), pp.147-153.

6. Navarro, V. et al., Citalopram versus nortriptyline in late-life depression: a 12-week randomized single-blind study, 2001, Acta Psychiatrica Scandinavica, 103(6), pp.435-440.

7. Allard, P. et al., Efficacy and tolerability of venlafaxine in geriatric outpatients with major depression: a double-blind, randomised 6-month comparative trial with citalopram, 2004, International Journal of Geriatric Psychiatry, 19(2), pp.1123-1130.

8. Klysner, R. et al., Efficacy of citalopram in the prevention of recurrent depression in elderly patients: a placebo-controlled study of maintenance therapy, (2002), The British Journal of Psychiatry: The Journal of Medical Science, 181(1), pp.29-35

**Fig. 1 (abstract A42).**
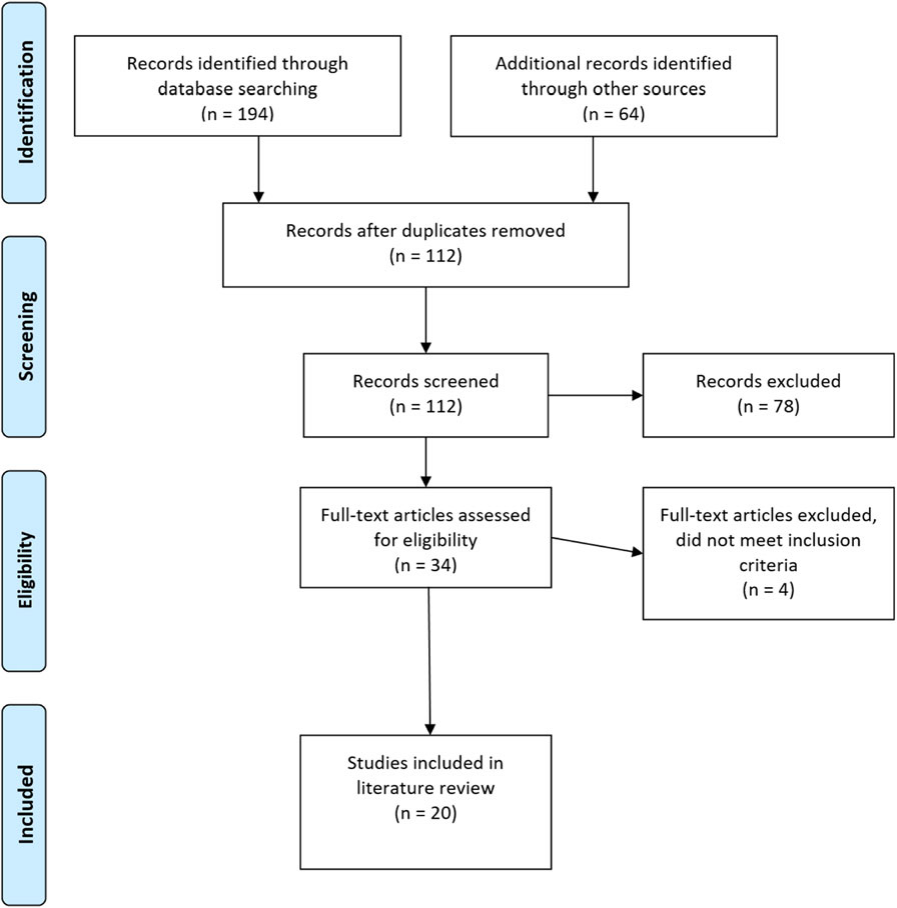
PRISMA Flow diagram; created using http://prisma-statement.org/PRISMAStatement/FlowDiagram

**Table 1 (abstract A42).** Summary of Citalopram treatment in the elderly

Reference	Study design	Result	Comment
Lavretsky et al. ^[4]^	Double-blind, placebo-controlled	Improved HAMD from baseline	Greater dose led to greater remission
Kyle et al. ^[5]^	Double-blind, double-dummy, parallel-group	Improved mood and better tolerance	Compared citalopram with amitriptyline
Navarro et al. ^[6]^	Single-blind	Improved mood and better tolerance	Compared citalopram to nortriptyline
Allard et al. ^[7]^	Double-blind, parallel	Improvement in MADRS compared to baseline	Venlafaxine as efficacious as citalopram
Klysner et al. ^[8]^	Open followed by double-blind	Improvement in MADRS	Citalopram associated with lower rates of recurrence

